# Calcium Influx Rescues Adenylate Cyclase-Hemolysin from Rapid Cell Membrane Removal and Enables Phagocyte Permeabilization by Toxin Pores

**DOI:** 10.1371/journal.ppat.1002580

**Published:** 2012-04-05

**Authors:** Radovan Fiser, Jiri Masin, Ladislav Bumba, Eva Pospisilova, Catherine Fayolle, Marek Basler, Lenka Sadilkova, Irena Adkins, Jana Kamanova, Jan Cerny, Ivo Konopasek, Radim Osicka, Claude Leclerc, Peter Sebo

**Affiliations:** 1 Faculty of Science, Charles University, Prague, Czech Republic; 2 Institute of Microbiology of the ASCR, v.v.i., Prague, Czech Republic; 3 Institut Pasteur, Paris, France; 4 INSERM U1041, Paris, France; 5 Institute of Biotechnology of the ASCR, v.v.i., Prague, Czech Republic; University of Illinois, United States of America

## Abstract

*Bordetella* adenylate cyclase toxin-hemolysin (CyaA) penetrates the cytoplasmic membrane of phagocytes and employs two distinct conformers to exert its multiple activities. One conformer forms cation-selective pores that permeabilize phagocyte membrane for efflux of cytosolic potassium. The other conformer conducts extracellular calcium ions across cytoplasmic membrane of cells, relocates into lipid rafts, translocates the adenylate cyclase enzyme (AC) domain into cells and converts cytosolic ATP to cAMP. We show that the calcium-conducting activity of CyaA controls the path and kinetics of endocytic removal of toxin pores from phagocyte membrane. The enzymatically inactive but calcium-conducting CyaA-AC^−^ toxoid was endocytosed *via* a clathrin-dependent pathway. In contrast, a doubly mutated (E570K+E581P) toxoid, unable to conduct Ca^2+^ into cells, was rapidly internalized by membrane macropinocytosis, unless rescued by Ca^2+^ influx promoted *in trans* by ionomycin or intact toxoid. Moreover, a fully pore-forming CyaA-ΔAC hemolysin failed to permeabilize phagocytes, unless endocytic removal of its pores from cell membrane was decelerated through Ca^2+^ influx promoted by molecules locked in a Ca^2+^-conducting conformation by the 3D1 antibody. Inhibition of endocytosis also enabled the native *B. pertussis*-produced CyaA to induce lysis of J774A.1 macrophages at concentrations starting from 100 ng/ml. Hence, by mediating calcium influx into cells, the translocating conformer of CyaA controls the removal of bystander toxin pores from phagocyte membrane. This triggers a positive feedback loop of exacerbated cell permeabilization, where the efflux of cellular potassium yields further decreased toxin pore removal from cell membrane and this further enhances cell permeabilization and potassium efflux.

## Author Summary

The adenylate cyclase toxin (CyaA) of pathogenic *Bordetellae* eliminates the first line of host innate immune defense by inhibiting the oxidative burst and complement-mediated opsonophagocytic killing of bacteria. The toxin penetrates myeloid phagocytes, such as neutrophil, macrophage or dendritic cells, and subverts their signaling by catalyzing a rapid and massive conversion of intracellular ATP to the key signaling molecule cAMP. In parallel, the toxin forms cation-selective pores and permeabilizes the cytoplasmic membrane of phagocytes. This so-called ‘hemolysin’ activity synergizes with the enzymatic AC activity of CyaA in promoting apoptotic or necrotic cell death, depending on the toxin dose. Moreover, the pore-forming activity promotes activation of NALP3 inflammasome and release of interleukin IL-1β. We show here that the capacity of CyaA to permeabilize phagocytes depends on its ability to mediate influx of extracellular calcium ions into cells. This enables bystander CyaA pores to escape rapid macropinocytic removal from cell membrane and exacerbate the permeabilization of cells. These observations set a new paradigm for the mechanism of action of pore-forming RTX leukotoxins on phagocytes.

## Introduction

By instantaneously disrupting bactericidal functions of host phagocytes, the adenylate cyclase toxin-hemolysin (CyaA, ACT, or AC-Hly) plays a major role in virulence of pathogenic *Bordetellae* [1]. The toxin rapidly paralyzes phagocytes [1], [2] by translocating across their cytoplasmic membrane an N-terminal adenylate cyclase enzyme (AC) domain (∼400 residues) that binds cytosolic calmodulin and converts ATP to a key signaling molecule, cAMP [1]. In parallel, the multidomain ∼1300 residues-long RTX (Repeat in ToXin) cytolysin moiety of CyaA acts independently as a pore-forming leukotoxin and hemolysin [1]. This employs a hydrophobic pore-forming domain (residues 500 to 700), a domain with covalently palmitoylated lysine residues 860 and 983, and a typical calcium-binding RTX repeat domain within the last 700 residues of CyaA that accounts for receptor binding [1]. CyaA can oligomerize into small cation-selective pores that mediate efflux of cytosolic potassium ions from cells [3]–[6], eventually provoking colloid-osmotic cell lysis [7]–[9]. This activity synergizes with cytotoxic signaling of the translocated AC enzyme in bringing about the final cytotoxic action of CyaA [8], [9].

The structure of CyaA in target membrane remains unknown. Accumulated indirect evidence strongly suggests that the cell-invasive AC and the pore-forming activities are associated with distinct subpopulations of CyaA conformers within target cell membrane. Indeed, translocation of the AC domain across cellular membrane and the pore-forming activity of CyaA can be dissociated by temperature, calcium concentrations, or altered acylation [5], [10], [11]. Moreover, the balance between the two activities can be largely shifted in either direction by specific residue substitutions. Several CyaA variants with strongly enhanced pore-forming activity and reduced or nil capacity to deliver the AC domain into cells could be generated. Recently, a CyaA defective in formation of toxin pores but intact in AC delivery into cells could also be constructed [3], [6], [12]. Summarizing this evidence, a model was proposed that predicts the co-existence of two distinct toxin conformers inside target cell membrane. These would operate in parallel and would employ the same segments of the hydrophobic domain in an alternative manner. One conformer would insert into cell membrane as toxin translocation precursor that accomplishes delivery of the AC domain into cells (cell-invasive activity). The second conformer would account for formation of oligomeric CyaA pores [3], [6], [12].

The primary targets of CyaA appear to be host myeloid phagocytes, to which the toxin binds through their α_M_β_2_ integrin, known as CD11b/CD18, CR3 or Mac-1 [13]. Recently, we could show that AC translocation into cells occurs in two steps. First, the membrane–inserted translocation precursors of CyaA generate a calcium-conducting path across cell membrane and mediate influx of extracellular calcium ions into cells [14]. The incoming Ca^2+^ than activates calpain-mediated processing of the talin tether of CD11b/CD18, which triggers relocation of the toxin-receptor complex into cholesterol-rich membrane lipid rafts. There the translocation of the AC domain into cytosol of cells across the cytoplasmic membrane of phagocytes is completed [15]. Unlike for most other enzymatic toxins, the capacity of CyaA to deliver its AC enzyme component into target cells does not depend on receptor-mediated endocytosis. Instead, the AC domain translocates into cytosol directly across the cytoplasmic membrane of phagocytes. Indeed, inhibitors of endocytic pathways interfere neither with translocation of the AC domain into cells and elevation of cytosolic cAMP, nor with AC domain-mediated delivery of inserted CD8^+^ T cell epitopes into the cytosol of antigen presenting cells [16]–[18]. Clathrin-dependent endocytic uptake of CyaA together with its receptor CD11b/CD18 has, however, previously been observed [19], [20] and it was found to account for the capacity of CyaA-AC^−^ toxoids to deliver cargo antigens into dendritic cells for endosomal processing and MHC II-restricted presentation to CD4^+^ T cells [18].

Therefore, we analyzed here the mechanism of endocytic uptake of CyaA from cell membrane and its relevance for toxin action. We show that by conducting calcium ions across the target membrane into cytosol of cells, the translocating CyaA molecules control the rate of toxin pore removal from cellular membrane and thereby support the permeabilization of phagocytes.

## Results

### Functional integrity of hydrophobic domain determines the path of endocytic trafficking of CyaA-AC^−^ toxoid

Recently we found that despite binding to CD11b/CD18, the CyaA constructs lacking the hydrophobic domain failed to deliver a fused MalE antigen for presentation to CD4^+^ T lymphocytes by dendritic cells (Figure S1). To address the role of functional integrity of CyaA in its endocytic trafficking, we employed live microscopy imaging.

To avoid the interference of massive phagocyte ruffling and death from ATP depletion and cAMP signaling provoked by enzymatically active CyaA at concentrations required for imaging [8], [21], we used fluorescently labeled and enzymatically inactive CyaA-AC^−^ toxoids. To enable assessment of their capacity to deliver antigens for presentation on MHC class I and II molecules, the toxoids were further tagged by insertion of a CD8^+^ T-cell epitope (SIINFEKL) from ovalbumin at position 336 and of a MalE CD4^+^ T-cell epitope (NGKLIAYPIAVEALS) at position 108 of the AC domain, respectively. Trafficking of such tagged dCyaA was then compared to that of a doubly mutated CyaA-E570K+E581P-AC^−^ toxoid (dCyaA-KP) that carried a combination of debilitating substitutions of glutamate residues at positions 570 and 581 within the pore-forming domain. That construct was previously shown to retain a full capacity to bind the toxin receptor CD11b/CD18, without being able to conduct calcium ions into cells, to associate with lipid rafts and translocate the AC domain across cell membrane, or to form oligomeric CyaA pores within cellular membrane, respectively [3], [15].

**Figure 1:**
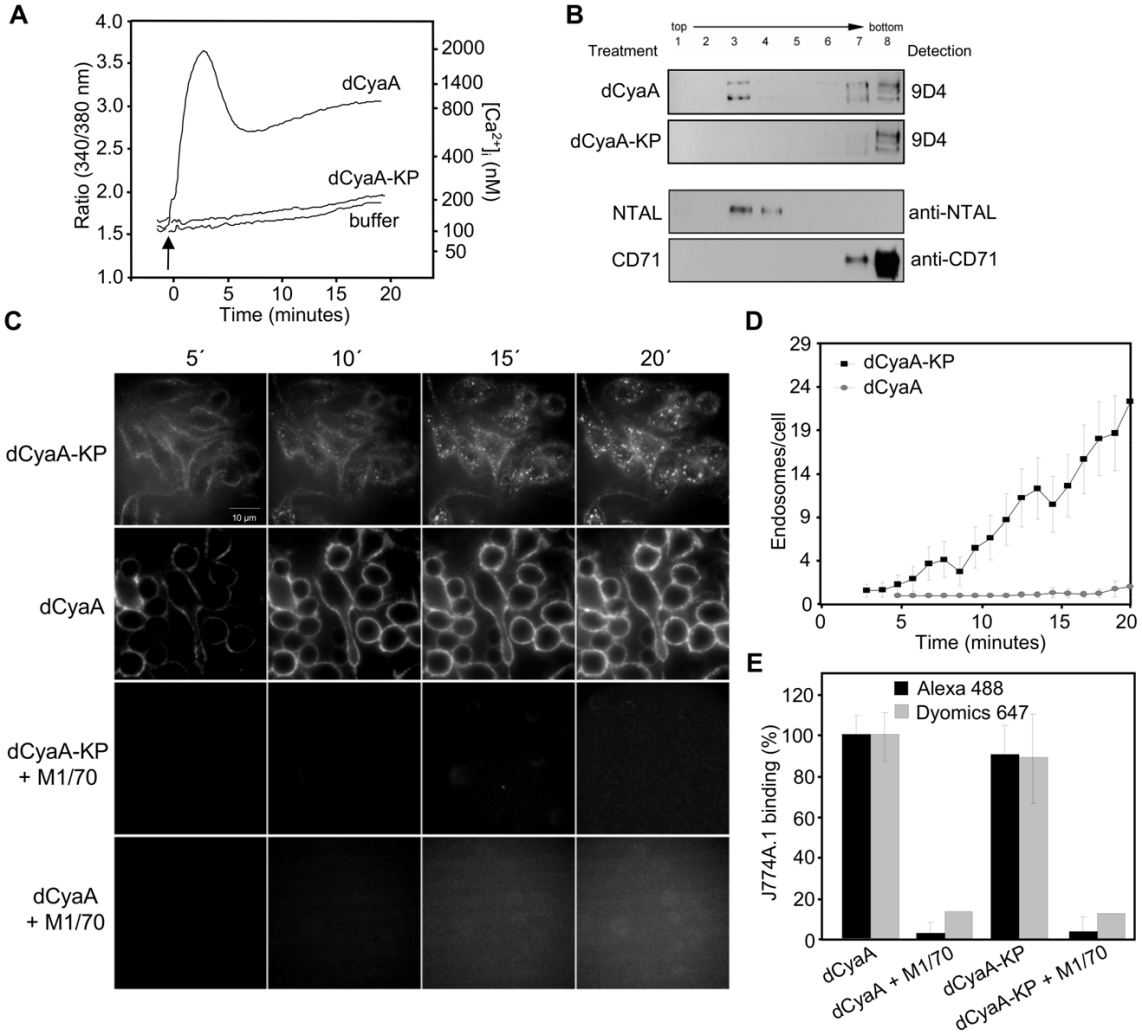
dCyaA-KP toxoid unable to promote calcium influx and relocate into lipid rafts is rapidly taken up by J774A.1 cells. (A) J774A.1 cells were loaded with the calcium probe Fura-2/AM at a 3 µM final concentration at 25°C for 30 min. After washing in HBSS medium, toxoid variants (3 µg/ml) or buffer were added at time zero (indicated by arrow) and time course of calcium entry into cytosol of cells (elevation of [Ca^2+^]_i_) was followed by spectrofluorimetry [14]. (B) J774A.1 cells were incubated for 10 minutes with 500 ng/ml of dCyaA or dCyaA-KP and detergent-resistant membrane microdomains (DRMs) were extracted with cold Triton X-100, separated by flotation in sucrose density gradient and probed in Western blots with the 9D4 antibody. The DRM fractions were defined as fractions enriched in the lipid raft marker NTAL. The transferrin receptor CD71 was used as a non-raft marker that remained in the bottom fractions of the gradient. (C) J774A.1 cells grown on glass bottom microwell dishes were incubated for 20 minutes at 37°C with 5 µg/ml of Dyomics 647-labeled dCyaA or Alexa Fluor 488-labeled dCyaA-KP proteins in the presence or absence of the anti-CD11b MAb M1/70 (20 µg/ml). Endocytic uptake of toxoids was analyzed at indicated time points by live cell imaging using an Olympus Cell^R^ IX 81 microscope with a 60×/1.35 oil objective (UPlanSApo). (D) The numbers of endosomes localized in cytoplasm of cells and loaded with dCyaA or dCyaA-KP were counted as described in detail in the legend to Supplementary figure S2, using a script based on WCIF ImageJ software (http://rsb.info.nih.gov/ij, http://www.uhnresearch.ca/facilities/wcif/imagej). The plot shows mean values plus standard deviations, as calculated on 20 to 40 cells per time point for one representative experiment out of three performed (n = 3). (E) J774A.1 cells (3×10^5^) were incubated for 30 min on ice at three different toxoid concentrations within the linear range of the dose-response curve (0.5, 1 and 5 µg/ml) and in the presence or absence of the anti-CD11b monoclonal antibody M1/70 (20 µg/ml, 15 min of preincubation of cells). Binding to cells was assessed as cell-associated toxoid fluorescence by FACS analysis. The % of toxoid binding at each concentration was calculated, taking the value for dCyaA toxoid in the absence of M1/70 as 100%. The average ± standard deviations are shown.

As shown in Figure 1, insertion of the epitope tags and fluorescent labeling did not alter the expected properties of the dCyaA toxoid, which elevated [Ca^2+^]_i_ in J774A.1 cells (Figure 1A) and relocated into lipid rafts in J774A.1 membrane (Figure 1B). As also expected, the dCyaA-KP toxoid lacked all these activities. When examined by live cell imaging, the two fluorescently labeled toxoids exhibited intriguingly different kinetics and patterns of endocytic uptake. As documented by representative images in Figure 1C and quantified in Figure 1D, dCyaA was detected predominantly within the plasma membrane, or in fluorescent vesicles located beneath the membrane of J774A.1 cells throughout the 20 minutes of incubation at 37°C (Video S1). In contrast, dCyaA-KP was taken-up much faster and accumulated massively within endocytic vesicles dispersed through the cytosol of cells already within 5 minutes from toxoid addition. The dCyaA-KP toxoid was then almost completely removed from cell membrane within 10 minutes (Figure 1C, Video S2), as quantified by counting of the intracellularly located fluorescent vesicles for a set of inspected cells (Figure 1D, see Figure S2 for the method). Identical results were observed upon swapping of the fluorescent labels (not shown), thus excluding the impact of the used dyes on toxoid trafficking. While the two toxoids entered into quite different endocytic pathways, the uptake of both was receptor-mediated and depended on toxoid binding to CD11b/CD18. Blocking of the receptor by the M1/70 antibody abrogated, indeed, cell binding and endocytosis of both toxoids, as documented in Figure 1C and Figure 1E.

**Figure 2:**
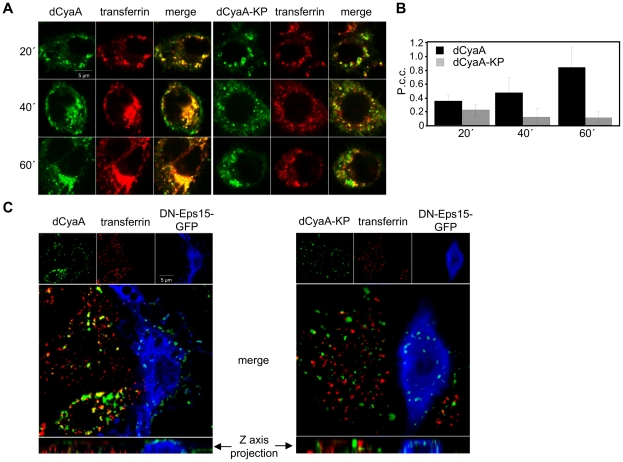
Intact dCyaA toxoid is endocytosed by a clathrin-dependent mechanism. (A) J774A.1 cells were incubated for 20, 40 and 60 minutes with 1 µg/ml of Alexa Fluor 488-labeled dCyaA or dCyaA-KP. For simultaneous visualization of early and recycling endocytic compartments, cells were co-incubated at 37°C for the last 20 minutes with Dyomics 547-labeled transferrin (10 µg/ml). Cells were washed in cold PBS, fixed with 4% PFA, and observed using a Leica confocal microscope TCS SP2 with a 63×/1.40 oil objective (HCX PL APO). (B) Co-localization of toxoids with transferrin was quantified using ImageJ software as described in details in Materials and Methods. (C) Murine RAW 264.7 macrophages transfected by dominant-negative mutant (DIII) of Eps-15 protein fused to GFP (blue) were incubated at 37°C for 30 min with the dCyaA or dCyaA-KP toxoids (1 µg/ml) labeled with Dyomics-647 (green). Dyomics 547-labeled transferrin (10 µg/ml) was added for the last 10 minutes of incubation (red), cells were washed with 0.1 M sodium acetate, 150 mM NaCl, pH 3.5 to remove surface-associated transferrin, fixed and observed with Olympus CellR microscope using a 100× oil immersion objective (N.A. 1.3). The calculated values of Pearson's correlation coefficients for transferrin (Dyomics 547) and toxoid (Dyomics 647) in non-transfected cells were 0.319 and 0.067 for dCyaA and dCyaA-KP, respectively.

To characterize the differing uptake pathways, we next examined the co-localization of the toxoids with transferrin, a marker of clathrin-dependent endocytosis [22]. As shown by representative images in Figure 2A and quantified by Pearson's correlation coefficients (P.c.c.) for compared channels in Figure 2B, the co-localization of dCyaA with transferrin (Dyomics 547) increased in time and was near-complete after 60 minutes of co-incubation with cells. In contrast, a weak and progressively decreasing co-localization of dCyaA-KP with transferrin was observed over the same time interval. Hence, while dCyaA was transiting through the early and/or recycling compartment together with transferrin, the dCyaA-KP toxoid took a different pathway.

**Figure 3:**
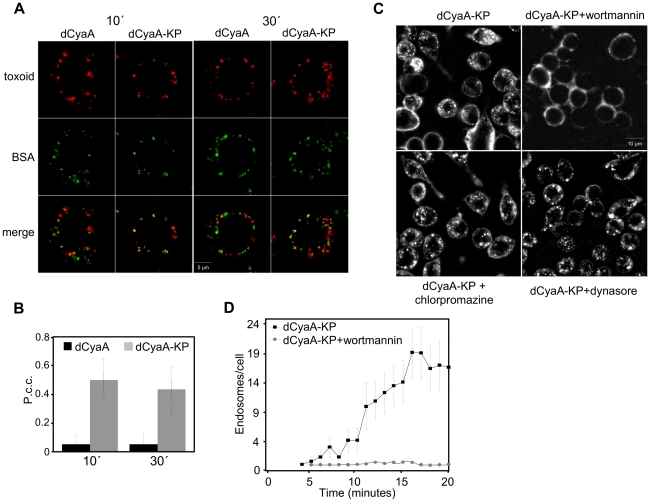
Inactive dCyaA-KP toxoid is endocytosed by a rapid macropinocytic pathway. (A) J774A.1 macrophages were incubated with BSA-Dyomics 547 (50 µg/ml) together with Alexa Fluor 488-labeled dCyaA or dCyaA-KP (1 µg/ml), respectively. After 10 and 30 minutes, the cells were washed, fixed and observed as above. (B) Co-localization of toxoids with BSA was quantified using ImageJ software as described in details in Materials and Methods. (C) J774A.1 cells were incubated for 30 minutes in HBSS with wortmannin (1 µg/ml), dynasore (40 µM) or chlorpromazine (5 µg/ml), before 5 µg/ml of Alexa Fluor 488-labeled dCyaA-KP was added and time course of toxoid uptake was followed by live cell imaging and (D) quantified as above (n = 3).

To corroborate this observation, the uptake of the two toxoids was examined in RAW 264.7 macrophages expressing a dominant negative variant of the EPS-15 protein (DN EPS-15-GFP, DIII), which selectively interferes with clathrin-dependent endocytosis [23]. As documented by a representative image in Figure 2C (left), the accumulation of transferrin and dCyaA at intracellular sites was abrogated in DN EPS-15-GFP-transfected cells (blue) and no co-localization of cell-associated dCyaA with transferrin was observed upon removal of surface-associated transferrin with a low pH buffer wash. As further documented by the z-axis projections, few if any intracellular vesicles containing dCyaA were observed inside DN EPS-15-GFP-transfected cells and the membrane-associated dCyaA was located exclusively inside fluorescent patches on cell surface. In striking contrast, the endocytic uptake of dCyaA-KP was unaffected in DN EPS-15-GFP-transfected cells and intracellular vesicles containing dCyaA-KP were clearly observed in the z-axis projections (Figure 2C, right panel). Neither of the two toxoids exhibited any co-localization with caveolin-1 (Figure S3). By difference to dCyaA, however, the dCyaA-KP exhibited a strong co-localization with soluble fluorescent BSA serving as a fluid phase uptake marker. As also shown in Figure 3C and quantified in Figure 3D, the kinetics of dCyaA-KP endocytic uptake was strongly decelerated upon pretreatment of cells with wortmannin (1 µM), a PI3 kinase inhibitor blocking macropinocytosis but not micropinocytosis [24]. In contrast, no inhibition of dCyaA-KP uptake was observed upon treatment with dynasore or chlorpromazine, the inhibitors of clathrin-dependent endocytosis [25], [26]. It can, hence, be concluded that dCyaA was endocytosed through a decelerated clathrin-dependent pathway, while dCyaA-KP was internalized with the cytoplasmic membrane by a macropinocytic mechanism.

### Macropinocytic uptake yields faster toxoid degradation and reduced delivery of antigenic cargo for MHC II-restricted presentation

Clathrin-dependent endocytosis was previously shown to enable dCyaA-mediated delivery of model antigens into CD11b^+^ dendritic cells for endosomal processing and MHC class II-restricted presentation to CD4^+^ T cells [18]. Therefore, we examined whether altered endocytic trafficking affected the antigen delivery capacity of the dCyaA-KP toxoid.

**Figure 4:**
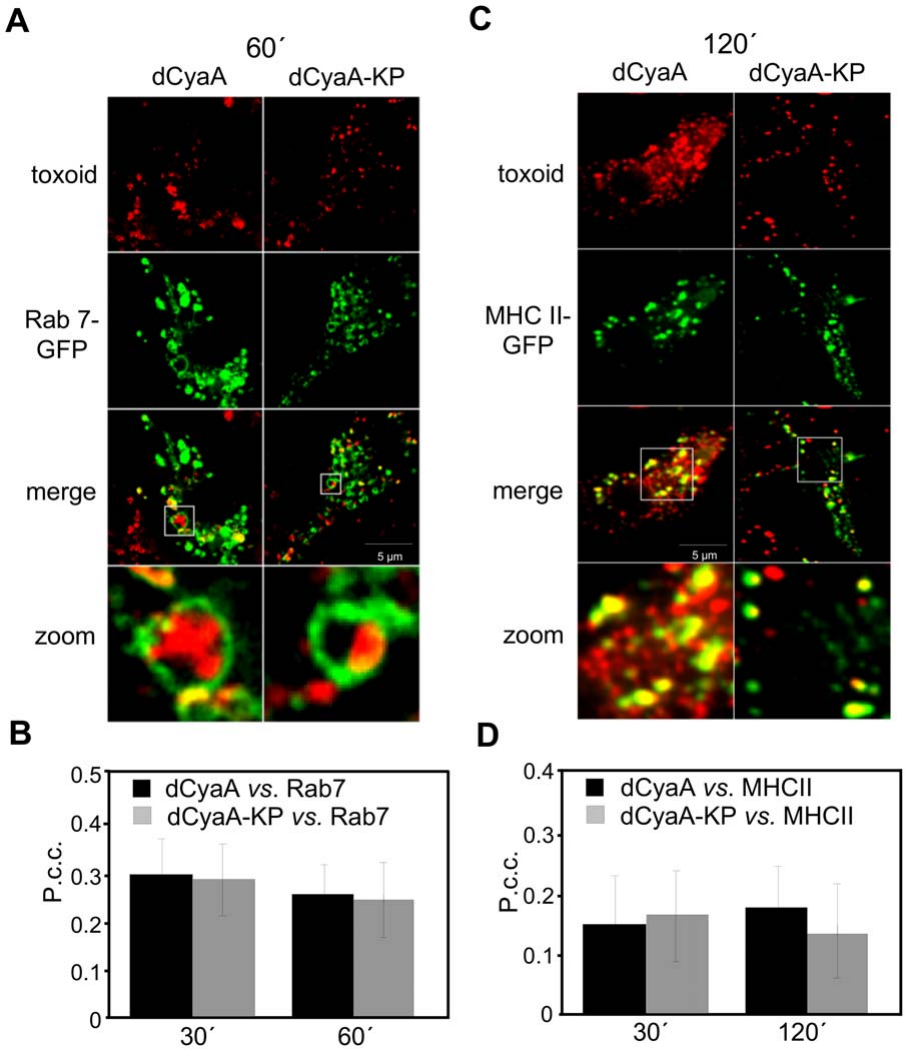
Both toxoids end up in MHC II-positive organelles. Transfected RAW 264.7 macrophages expressing Rab-7-EGFP were incubated for 60 minutes with Dyomics 647-labeled toxoids (1 µg/ml), washed, fixed and analyzed by live cell imaging. (B) Co-localization of toxoids with Rab-7-EGFP was quantified using ImageJ software. (C) Cells from MHC Class II/EGFP knock-in mice (green) were incubated at 37°C in DMEM without serum for 2 hours with 1 µg/ml of Dyomics 647-labeled (red) dCyaA or dCyaA-KP. Cells were fixed in 4% PFA and mounted in Mowiol. Images were captured using an inverted Olympus Cell^R^ microscope equipped with a 60×/1.35 oil immersion objective (UPlanSApo). (D) Co-localization of toxoids with MHC II/EGFP was quantified using ImageJ software.

As documented in Figure 4A and quantified in Figure 4B, following one hour of incubation with RAW 264.7 macrophages expressing Rab-7-EGFP, fluorescently labeled material derived from either of the toxoids accumulated within organelles positive for the lysosomal/late endosomal marker Rab7. dCyaA and dCyaA-KP toxoid-derived fluorescence also co-localized to the same extent with the MHC II–EGFP molecules in a subset of intracellular vesicles of bone marrow derived dendritic cells from a MHC Class II–EGFP knock-in mice, within 120 minutes of incubation (Figure 4C, 4D). Despite entering cells by different endocytic pathways, hence, the two toxoids or their fragments reached similar acidic endosomes.

**Figure 5:**
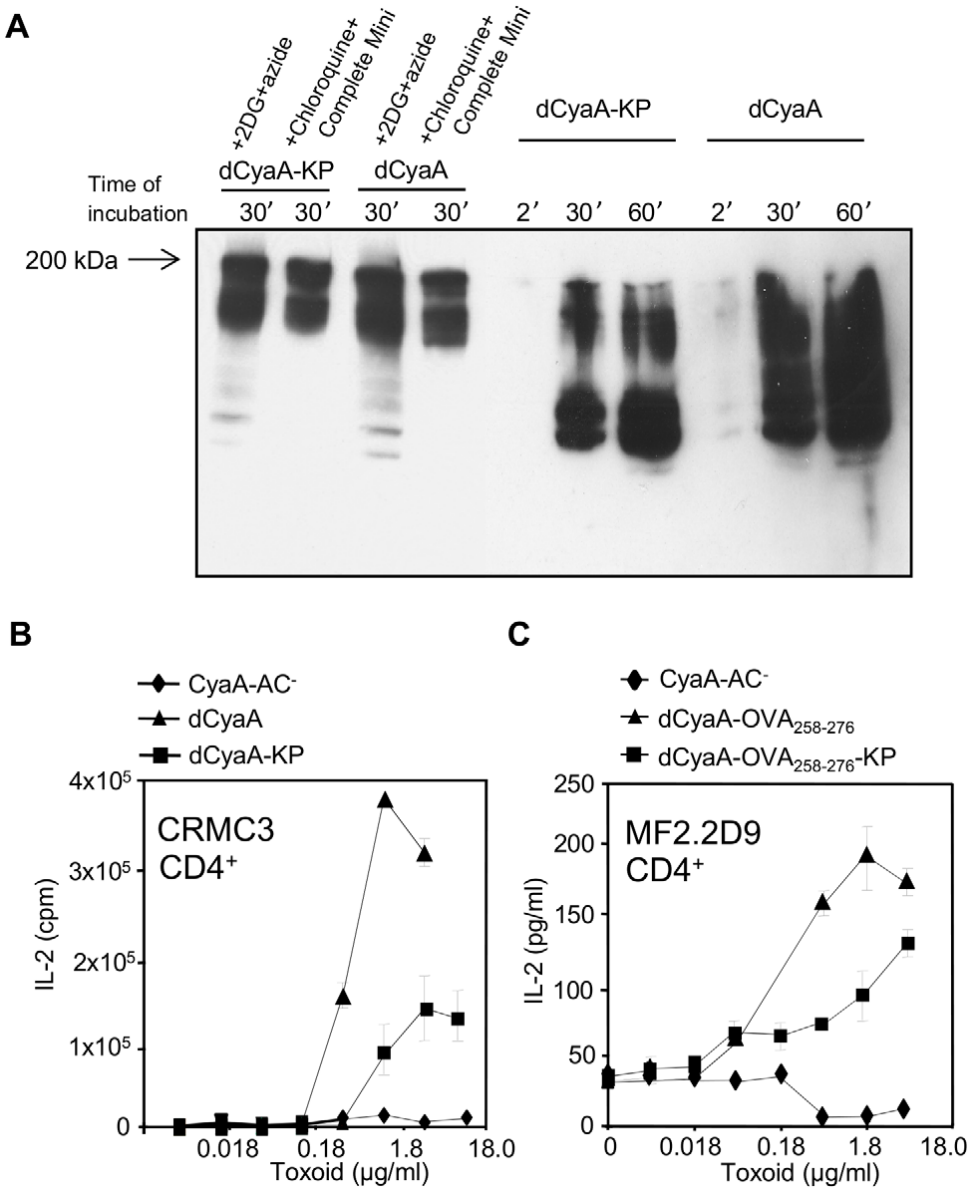
Macropinocytosed dCyaA-KP toxoid is rapidly degraded and its capacity to deliver epitopes for presentation on MHC class II molecules is compromised. (A) 10^6^ J774A.1 cells were preincubated for 30 minutes at 37°C in 1 ml of D-MEM medium alone, or in D-MEM containing 1 mM chloroquine plus a cocktail of protease inhibitors (Complete Mini, Roche), or in D-MEM containing 10 mM 2DG and 0.01% of sodium azide, respectively. dCyaA or dCyaA-KP toxoids were added to a final concentration of 1 µg/ml and after continued incubation the cells were washed three times in ice-cold D-MEM at the indicated times and lyzed on ice during 30 minutes in TBS buffer containing 1% Triton X-100 and the protease inhibitor cocktail (Complete Mini, Roche). Cell nuclei were removed by centrifugation at 13,000 RPM for 10 min and supernatants of lyzed cells were separated by 7.5% SDS-PAGE. CyaA fragments were detected in Western blots using the 9D4 antibody recognizing the C-terminal RTX repeats of CyaA. The arrow indicates migration of full-length (undegraded) 200 kDa CyaA. (B) BMDC from C57BL/6 mice were pulsed with indicated concentrations of dCyaA or dCyaA-KP, or (C) with dCyaA-OVA_258–276_ or dCyaA-OVA_258–276_-KP and following medium disposal, the MalE-specific hybridoma CRMC3 (B) or OVA_258–276_-specific hybridoma MF2.2D9 cells (C) were added, respectively. Secretion of IL-2 by antigen-stimulated CRMC3 hybridoma (B) was determined as proliferation of the IL-2-dependent CTL-L cell line and the results are expressed in cpm ± SE of duplicate samples. The concentration of IL-2 produced by MF2.2D9 cells upon antigenic stimulation (C) was determined by sandwich ELISA. A representative Western blot from 3 independent experiments and the averaged values from two independent antigen presentation experiments performed in duplicates (n = 4), are shown.

To test if faster macropinocytic uptake lead to alteration of dCyaA-KP processing, lyzates from toxoid-pulsed J774A.1 cells were probed in Western blots with the 9D4 MAb that recognizes C-terminal RTX repeats of CyaA. As shown in Figure 5A, comparable amounts of the ∼200 kDa forms of both toxoids and of their fragments were detected in lyzates of cells pretreated with inhibitors of endocytosis and protease inhibitors, like 0.01% sodium azide plus 10 mM 2-deoxy glucose (2DG), or a protease inhibitor cocktail plus 1 mM chloroquine. As compared to dCyaA, however, a notably faster degradation dCyaA-KP occurred in uninhibited cells (Figure 5A), as judged from the detected amounts of smear corresponding to partially digested molecules. dCyaA-KP toxoid, hence, appeared to be degraded faster and more completely, albeit the final protease-resistant fragments of both toxoids appeared to be of similar size.

To examine if faster uptake and degradation affected the capacity of dCyaA-KP to deliver cargo epitopes for endosomal processing and MHC II-restricted presentation, the relative capacity of the two toxoids to deliver CD4^+^ T cell epitopes was assessed. As shown in Figure 5B by comparison to dCyaA, the dCyaA-KP toxoid exhibited an about ten-fold reduced capacity to deliver the MalE epitope for presentation by BMDCs to CRMC3 CD4^+^ T hybridoma cells [27]. To corroborate this observation, the MalE epitope was replaced by the OVA_258–276_ epitope recognized by MF2.2D9 CD4^+^ T hybridoma and new dCyaA and dCyaA-KP toxoids were produced. As shown in Figure 5C, however, a similarly reduced capacity to deliver the OVA_258–276_ epitopes for MHC II-restricted presentation was again observed with dCyaA-KP, as compared to dCyaA. This suggested that faster proteolytic destruction of the cargo epitope during trafficking through the macropinocytic-like pathway may have accounted for the reduced efficacy of dCyaA-KP in antigen delivery.

### Permeabilization of cells for calcium prevents rapid macropinocytic removal of the toxoid from cell membrane

**Figure 6:**
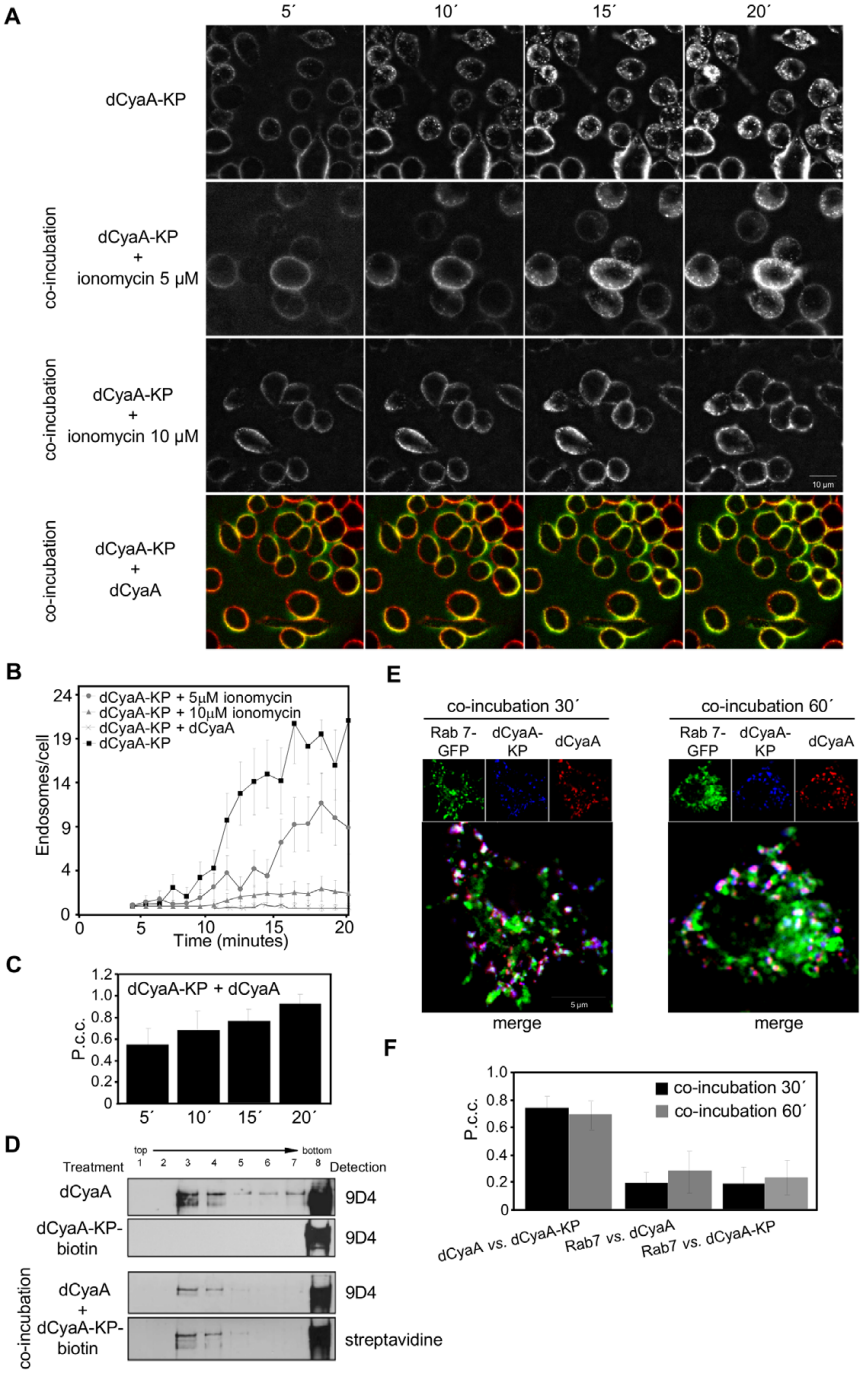
Calcium influx into J774A.1 macrophages decelerates endocytic uptake of the dCyaA-KP toxoid. (A) J774A.1 cells were incubated for 20 minutes at 37°C with 5 µg/ml of Dyomics 647-labeled dCyaA-KP alone, or in the presence of ionomycin (5 µM and 10 µM), or as a 1∶1 mixture with Alexa 488-labeled dCyaA. (B) Uptake of toxoids into cells was analyzed by live cell imaging as above and (C) P.c.c. values for the mixed sample of dCyaA and dCyaA-KP were calculated. (D) J774A.1 cells were incubated for 10 minutes with 500 ng/ml of dCyaA, dCyaA-KP-biotin or with 1∶1 mixture of dCyaA and dCyaA-KP-biotin toxoids. Detergent-resistant membrane microdomains (DRMs) were extracted, separated and probed as in Figure 1B. Biotin-labeled dCyaA-KP was detected with streptavidin HRP conjugate (GE Healthcare, Chalfont St. Giles, UK). (E) Transfected RAW 264.7 macrophages expressing Rab-7-EGFP were incubated for 30 and 60 minutes with 1 µg/ml of Dyomics 547 (dCyaA) and Dyomics 647-labeled toxoids (dCyaA-KP), washed, fixed and analyzed fluorescence imaging and (F) P.c.c. values were determined as in Figure 4.

To test whether the loss of capacity to mediate influx of Ca^2+^ ions into cells committed dCyaA-KP for the rapid macropinocytic uptake and subsequent degradation, the J774A.1 cells were incubated with dCyaA-KP in the presence of ionomycin, a calcium ionophore that permeabilizes cells for extracellular Ca^2+^. As documented in Figure 6A, Video S3, and as quantified in Figure 6B, respectively, internalization of dCyaA-KP into fluorescent intracellular vesicles was strongly delayed upon treatment of J774A.1 cells with 5 or 10 µM ionomycin in the presence of 1.9 mM Ca^2+^. As also shown in Figure 6A (Video S4) and quantified in Figure 6B and 6C, dCyaA-KP was redirected into a slower uptake pathway even more efficiently upon co-incubation at a 1∶1 ratio with intact dCyaA. This goes well with our previous observation that permeabilization of cells for Ca^2+^ *in trans* by intact dCyaA rescued in part the defect of a CyaA-KP construct and mobilized it into lipid rafts [15]. Upon co-incubation with dCyaA, a fraction of biotinylated dCyaA-KP protein was indeed found to float in sucrose gradients with detergent-resistant membrane (Figure 6D). Furthermore, following co-incubation with dCyaA, the dCyaA-KP appeared to be endocytosed with the same kinetics and through the same pathway as the intact toxoid (Videos S1 and S4). The two proteins co-localized within the same endocytic vesicles at 30 and 60 minutes of incubation with cells (Figure 6E, 6F). Hence, permeabilization of cells for Ca^2+^ by ionomycin or intact toxoid rescued dCyaA-KP from the rapid macropinocytic membrane uptake pathway and redirected it for decelerated and clathrin-dependent endocytosis.

### Calcium-induced deceleration of endocytic removal from plasma membrane enables CyaA hemolysin pores to permeabilize phagocytes

**Figure 7:**
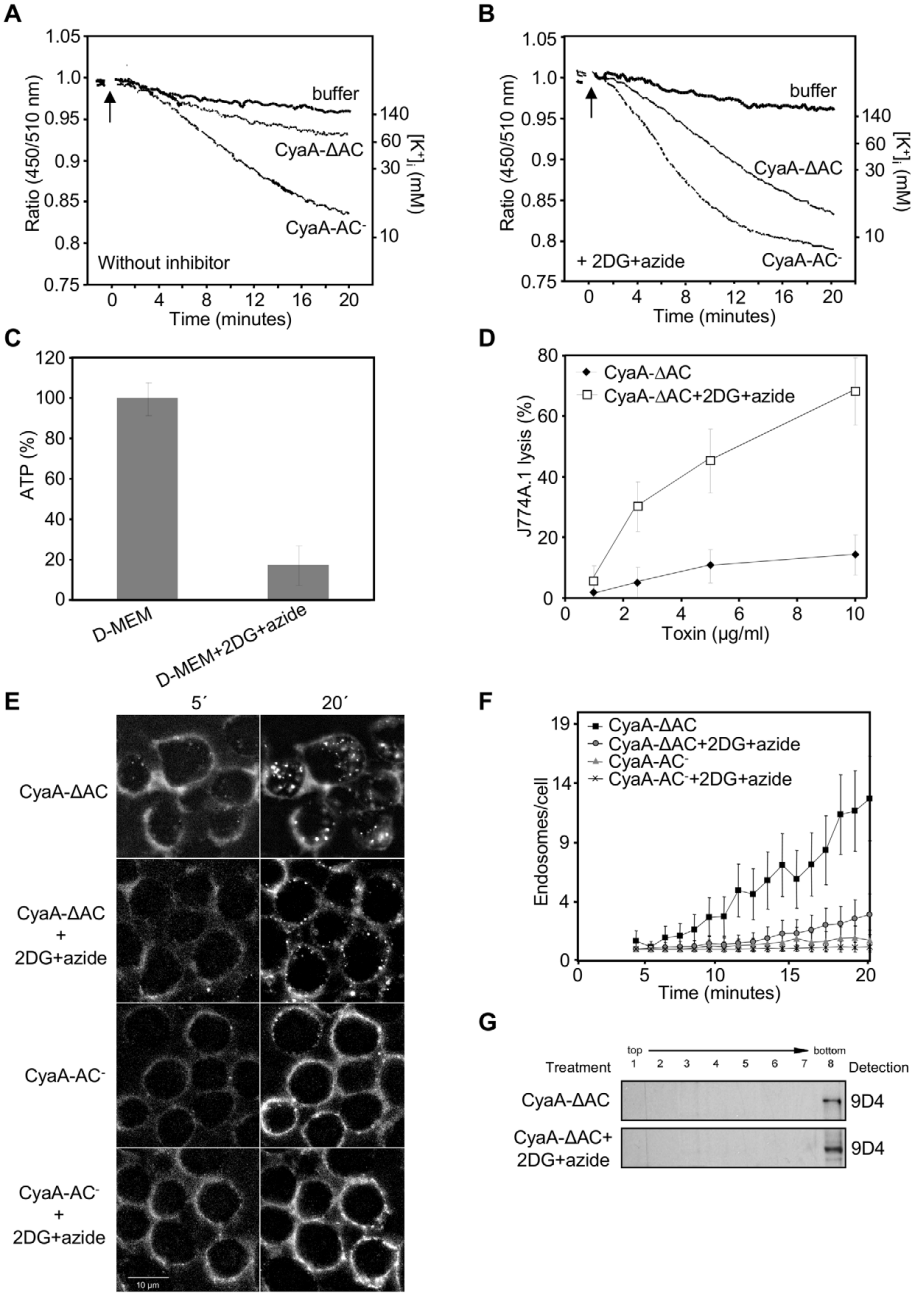
Rapid removal from the plasma membrane reduces toxoid-mediated K^+^ efflux from monocytes. (A) PBFI/AM loaded J774A.1 monocytes in HBSS buffer were exposed to 3 µg/ml of enzymatically inactive CyaA-AC^−^ toxoid, or to its CyaA-ΔAC variant, and K^+^ efflux at 25°C was monitored in time as described under Materials and Methods. (B) PBFI/AM loaded J774A.1 macrophages were preincubated for 15 minutes in HBSS buffer containing 0.01% sodium azide and 10 mM 2DG prior to addition of CyaA-AC^−^ or CyaA-ΔAC proteins and time course of K^+^ efflux was recorded. The shown curves are representative of six independent experiments. (C) 10^6^ J774A.1 cells were incubated for 15 minutes in D-MEM or in D-MEM containing the glycolysis inhibitor 2-deoxy-D-glucose (2DG, 10 mM) instead of glucose and 0,01% sodium azide. ATP content in cells was determined using the ATP Bioluminescence Assay Kit CLS II (Roche). (D) Prior to addition of the CyaA-ΔAC protein, 2×10^5^ J774A.1 macrophages were preincubated for 15 minutes at 37°C in D-MEM alone or in D-MEM medium without glucose and containing 0.01% sodium azide plus 10 mM 2DG. The extent of cell lysis was determined using the Cytotox 96 assay kit (Promega) as the amount of lactate dehydrogenase released into culture media in 2 hours. Average values from three independent experiments performed in duplicates are shown. (E) CyaA-ΔAC, in contrast to CyaA-AC^−^ is rapidly internalized into J774A.1 cells. J774A.1 cells were preincubated for 15 minutes in HBSS, or in glucose-free HBSS with 2DG and sodium azide, before 5 µg/ml of Fluor 488-labeled CyaA-ΔAC or CyaA-AC^−^ were added. Toxoid endocytosis was analyzed by live cell imaging and (F) Numbers of endosomes containing CyaA-ΔAC and CyaA-AC^−^ were determined as above. (G) J774A.1 cells were preincubated for 15 minutes in HBSS or in HBSS containing sodium azide and 2DG prior to addition of CyaA-ΔAC (500 ng/ml). Detergent-resistant membrane microdomains (DRMs) were extracted, separated and probed as in Figure 1B.

We have previously observed that the CyaA-ΔAC hemolysin construct lacking the AC domain of CyaA (Δ1–373) was unable to promote Ca^2+^ influx into monocytes and was essentially unable to provoke lysis of J774A.1 cells [14]. However, on planar lipid bilayers, or on sheep erythrocytes devoid of endocytic mechanisms, the CyaA-ΔAC hemolysin exhibited the same specific pore-forming and hemolytic activities as the full-length dCyaA (CyaA-AC^−^) [4]. We thus hypothesized that its inability to lyze J774A.1 cells might be due to rapid removal of the CyaA-ΔAC pores from the cytoplasmic membrane of J774A.1 cells. As shown in Figure 7A, the CyaA-ΔAC hemolysin elicited much slower efflux of cytosolic K^+^ from J774A.1 cells then the full-length toxoid. In cells exposed to 3 µg/ml of enzymatically inactive CyaA-AC^−^ a complete drop of cytosolic [K^+^]_i_ concentration down to 10 mM was reproducibly observed within 20 minutes, while in cells exposed to equal amounts of CyaA-ΔAC the [K^+^]_i_ only decreased to 80 mM (Figure 7A).

To determine if this was due to rapid removal of CyaA-ΔAC pores from cell membrane, we assessed the capacity of CyaA-ΔAC to elicit K^+^ efflux on cells with membrane trafficking inhibited upon preincubation in media containing 0.01% (w/w) sodium azide and 10 mM 2-deoxy-glucose (2DG). This treatment potentiated the cell-permeabilizing activity of full-length CyaA-AC^−^ toxoid (Figure 7A, 7B), but did not promote any significant K^+^ efflux from cells on its own despite causing an about five-fold drop of cellular ATP levels (Figure 7A, 7B and 7C). As further documented in Figure 7E and 7F (Videos S5 and S6), the strong inhibition of the CyaA-ΔAC uptake was accompanied by a steep increase of the specific capacity of CyaA-ΔAC to promote K^+^ efflux (Figure 7A and 7B) from cells and lyze J774A.1 monocytes (Figure 7D). It can thus be concluded that rapid macropinocytic internalization with cell membrane was strongly restricting the cell permeabilizing and cytolytic capacities of the CyaA-ΔAC hemolysin pores.

As further shown in Figure 7G, despite the prolonged persistence in the cytoplasmic membrane of cells upon inhibition of endocytosis, the CyaA-ΔAC hemolysin failed to associate with lipid rafts (compare CyaA-ΔAC panel in Figure 7G and upper dCyaA panel of Figure 1B for comparable toxoid loading). This goes well with our previous observation that mobilization into lipid rafts with CD11b/CD18 depends on the capacity of CyaA to conduct calcium ions into cells to activate talin cleavage by calpain [15]. More importantly, this result shows that CyaA pores can form outside of lipid rafts within the bulk phase of cell membrane.

**Figure 8:**
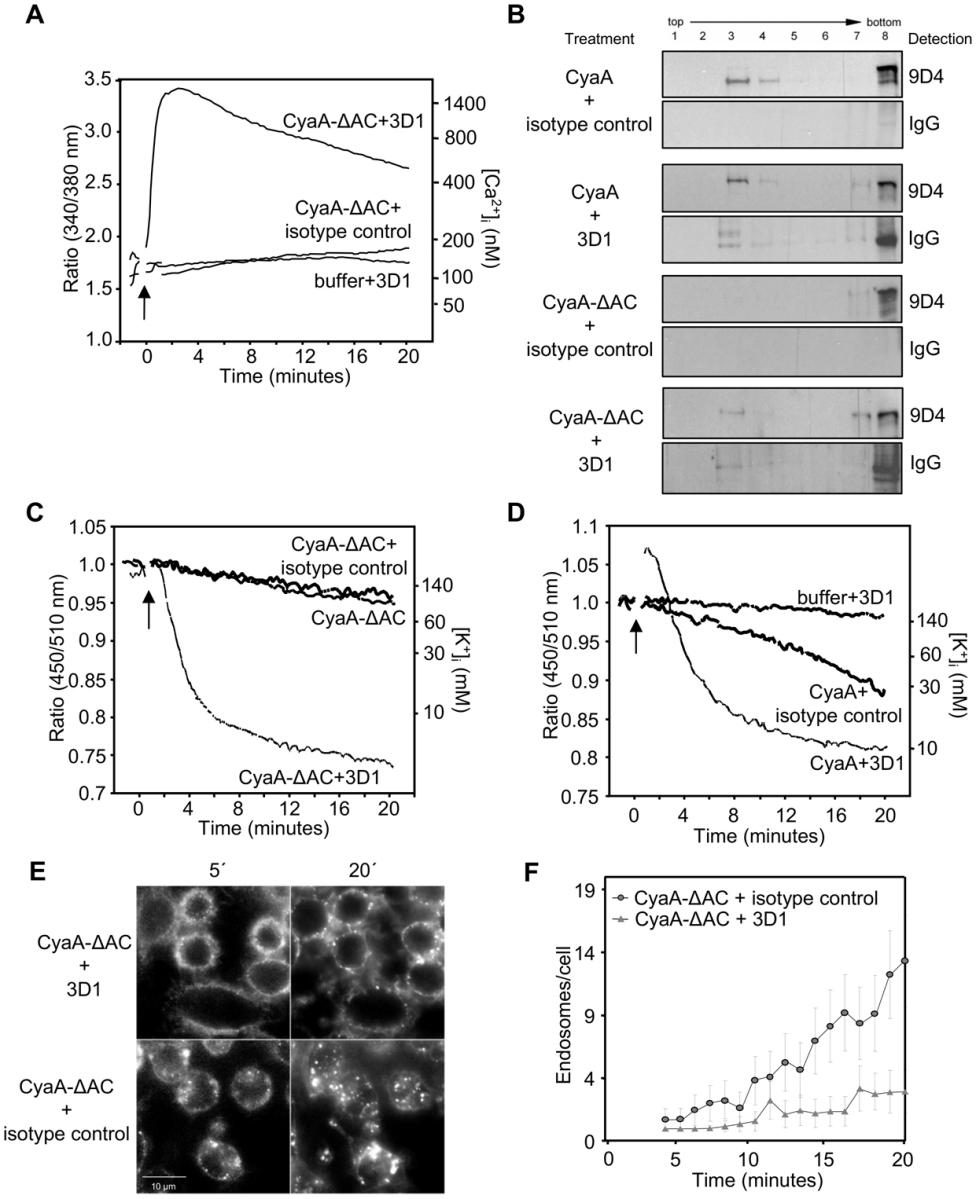
Influx of Ca^2+^ decelerates macropinocytic uptake of CyaA-ΔAC and enables cell permeabilization by hemolysin pores. (A) J774A.1 cells were loaded with Fura-2/AM and exposed to 3 µg/ml of CyaA-ΔAC (arrow) preincubated for 15 minutes at room temperature with 3D1, or with the isotype control antibody TU-01 (20 µg/ml) and Ca^2+^ influx was recorded. The shown curves are representative of three independent experiments. (B) J774A.1 cells were exposed for 10 minutes at 37°C to CyaA protein variants (500 ng/ml) preincubated with 3D1 or TU-01 (20 µg/ml) and cell lyzates were separated on sucrose density gradients as in Figure 1B. The 3D1 and isotype IgG1 mAbs were detected with anti-mouse IgG antibody. (C) 3 µg/ml of CyaA-ΔAC or of CyaA (D) were preincubated with 3D1 or TU-01 (20 µg/ml), added to PBFI/AM loaded J774A.1 macrophages and time course of K^+^ efflux from J774A.1 was recorded. The curves are representative of four independent experiments. (E) J774A.1 cells were exposed to 5 µg/ml of Alexa 488-labeled CyaA-ΔAC preincubated with 3D1 or TU-01 mAb (20 µg/ml). The time course of CyaA-ΔAC endocytosis was followed by live cell imaging and (F) quantified.

We next tested if Ca^2+^ influx induced *in trans* would delay removal of CyaA-ΔAC from cytoplasmic membrane and extend thereby its capacity to permeabilize J774A.1 cells. Mobilization of Ca^2+^ into cells with ionomycin, however, caused on its own a high unspecific leakage of K^+^ from J774A.1 cells (data not shown). Therefore, an alternative approach was used, exploiting the capacity of the 3D1 MAb to lock CyaA molecules in the conformation of membrane-inserted ‘translocation precursors’ that conduct calcium ions across the cytoplasmic membrane of cells [15]. CyaA-ΔAC was preincubated with 3D1, or with an isotype control IgG1 MAb (TU-01, 20 µg/ml) and the capacity of the CyaA-ΔAC to elicit K^+^ efflux from J774A.1 cells was assessed. As documented in Figure 8A, while the 3D1 MAb alone did not cause any elevation of [Ca^2+^]_i_, the binding of 3D1 conferred on CyaA-ΔAC the capacity to promote rapid influx of Ca^2+^ into monocytes. As shown in Figure 8B, this allowed association of detectable amounts of CyaA-ΔAC molecules with lipid rafts. More importantly, the capacity of CyaA-ΔAC to permeabilize J774A.1 cells for K^+^ efflux was strongly enhanced in the presence of 3D1 MAb and equaled the specific cell-permeabilizing activity of the intact and enzymatically active CyaA, as shown in Figure 8C and 8D. Furthermore, this enhancement of cell-permeabilizing activity was accompanied by a strong deceleration of endocytic uptake of CyaA-ΔAC (Figure 8E, 8F).

**Figure 9:**
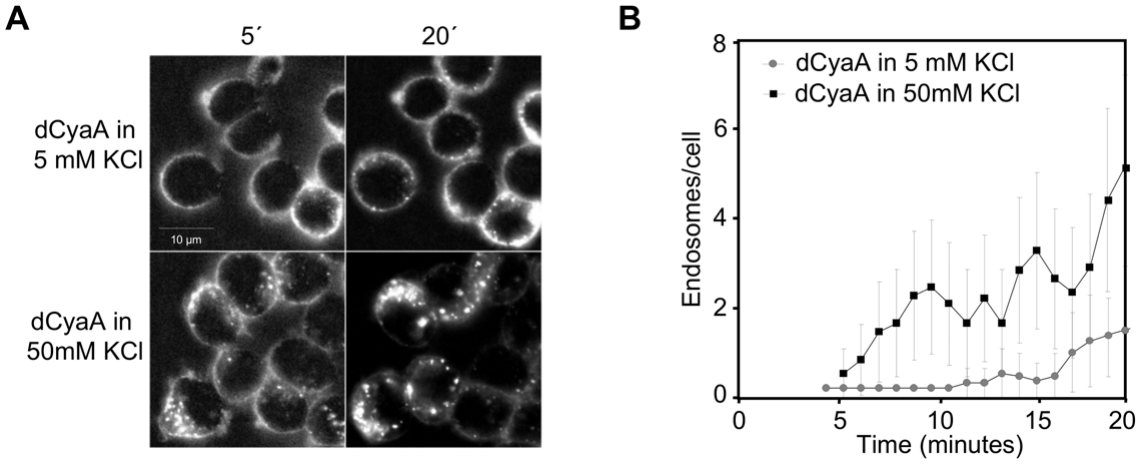
Potassium efflux delays endocytosis of dCyaA toxoid. A) J774A.1 cells were incubated for 20 minutes at 37°C with 5 µg/ml of Alexa 488-labeled dCyaA in HBSS containing 5 mM KCl or in HBSS containing 50 mM KCl. Uptake of dCyaA into cells was analyzed by live cell imaging and (B) numbers of endosomes in cell cytoplasm were quantified (n = 3).

To test the hypothesis that toxoid-induced K^+^ efflux itself contributed further deceleration of endocytosis, the clathrin-mediated uptake of dCyaA was examined in media supplemented with 50 mM potassium ions. As documented in Figure 9A and quantified in Figure 9B, the endocytosis of dCyaA was clearly accelerated when efflux of K^+^ from cells was counteracted by addition of potassium ions into media. Collectively, hence, these results show that CyaA-mediated Ca^2+^ influx rescues hemolysin pores from rapid macropinocytic uptake from cell membrane and thus extends their cell-permeabilizing and cytolytic action.

### Endocytosis controls also the cytolytic potency of enzymatically active CyaA

The pore-forming activity was previously shown to synergize with the ATP-depleting and cAMP-signaling activities of the cell-invasive AC domain of CyaA [8], [9]. Therefore, we examined to which extent does endocytic uptake modulate the cytotoxicity of fully active (AC^+^) toxin.

**Figure 10:**
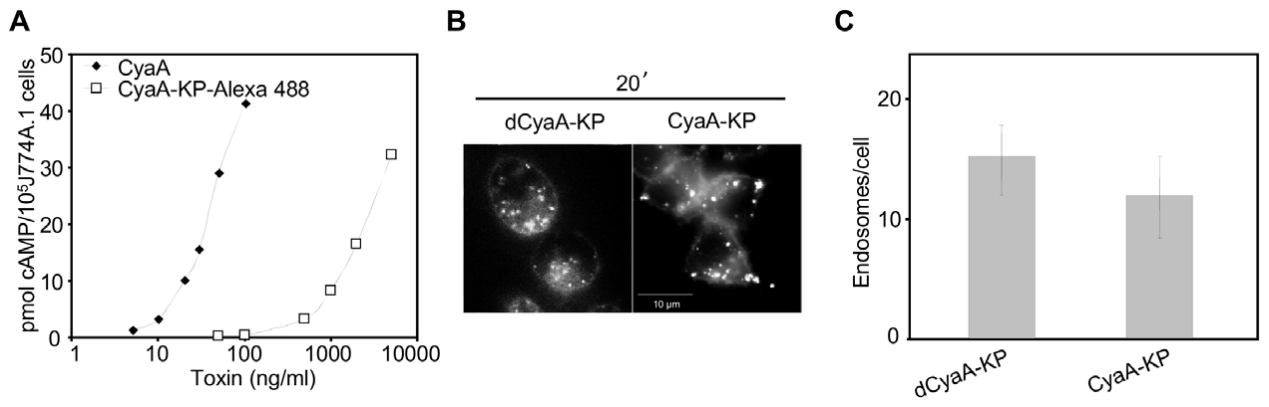
Endocytosis of CyaA is not affected by cAMP. (A) The capacity to translocate the AC domain across cytoplasmic membrane was assessed as the capacity to elevate cytosolic concentrations of cAMP. J774A.1 cells were incubated with CyaA or CyaA-KP-Alexa 488 for 30 minutes at 37°C and the amounts of accumulated cAMP were determined in cell lyzates. The shown curves are representative of two independent experiments performed in duplicates. (B) J774A.1 cells were incubated for 20 minutes at 37°C with 5 µg/ml of Alexa 488-labeled dCyaA-KP or CyaA-KP constructs, the protein uptake was analyzed by live cell imaging and (C) the numbers of endosomes in cytoplasm were quantified as above.

We assessed first the impact of cAMP accumulation on the uptake of CyaA, using a fluorescently labeled CyaA-KP construct (AC^+^) that exhibits only a residual capacity to deliver the AC enzyme into cells. Unlike active toxin, hence, CyaA-KP does not provoke cell death at the high concentrations (1 to 5 µg/ml) employed in live cell imaging. As shown in Figure 10A, at such concentrations the dCyaA-KP produced easily detectable cAMP amounts in cells, while the extent of its endocytic uptake was the same as that of the enzymatically inactive (AC^−^) dCyaA-KP toxoid (Figure 10B, 10C). Since both proteins were unable to mediate detectable Ca^2+^ influx into J774A.1 cells (ref. [15] and Figure 1B), it can be concluded that cAMP levels alone do not noticeably influence the rate of endocytic uptake of CyaA.

**Figure 11:**
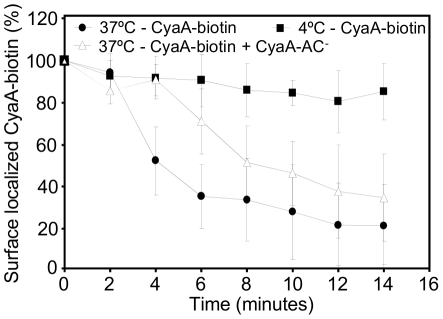
Ca^2+^ influx and K^+^ efflux promoted *in trans* decelerate the endocytic uptake of enzymatically active CyaA. J774A.1 macrophages (3×10^6^/ml) in D-MEM medium were incubated for 5 minutes at 37°C with 200 ng/ml of r*Ec*-CyaA-biotin, washed in D-MEM and further incubated at 4°C or 37°C, respectively, in the presence or absence of 1 µg/ml of unlabeled CyaA-AC^−^. At indicated time points cell-surface localized r*Ec*-CyaA-biotin *per* 3×10^5^ cells was detected with PE-conjugated Streptavidine (0.5 mg/ml) by FACS analysis (n = 3).

It was next important to characterize the rate of endocytic uptake of CyaA at as low toxin concentrations as that presumably encountered by host phagocytes during *Bordetella* infections. Therefore, the kinetics of CyaA endocytosis by J774A.1 cells was assessed by a flow cytometry assay measuring the amount of biotinylated CyaA that remains accessible to binding by streptavidin on cell surface. As shown in Figure 11, pulsing of J774A.1 cells for 5 minutes at 37°C with 200 ng/ml of CyaA-biotin yielded about 1 ng of toxin stably bound to 3×10^5^ washed cells, when kept on ice. In contrast, the amounts of surface-exposed CyaA-biotin decreased progressively upon transfer of cells to 37°C, with ∼80% of CyaA being removed from cell surface within 15 minutes. As also shown in Figure 11, the rate of CyaA-biotin uptake was reduced by about a factor of two in the presence of a 5-fold (non-saturating) excess of unlabeled CyaA-AC^−^ toxoid (1 µg/ml) that enhanced Ca^2+^ influx and K^+^ efflux across the membrane of J774A.1 cells [14].

**Figure 12:**
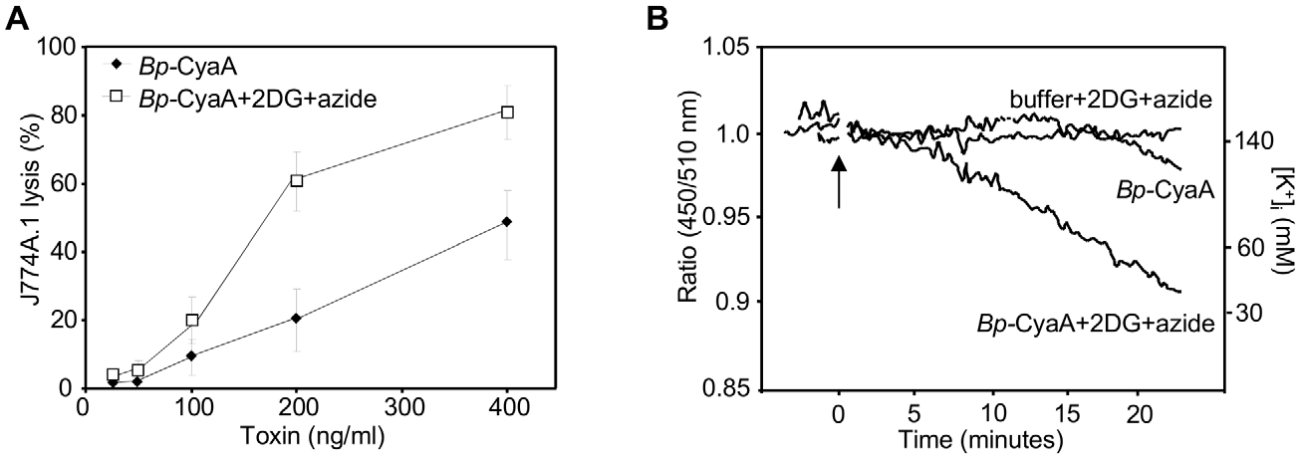
Endocytic removal from cell membrane controls the cytolytic potency of CyaA toxin. (A) 2×10^5^ J774A.1 macrophages were preincubated for 15 minutes in D-MEM medium alone or in D-MEM without glucose containing 0.01% sodium azide and 10 mM 2DG prior to addition of *Bp*-CyaA purified from *B. pertussis* 18323/pHSP9. The extent of cell lysis was determined as the amount of lactate dehydrogenase released into culture media in 2 hours, using the Cytotox 96 assay kit (Promega). The results represent the average of values obtained in three independent experiments performed in duplicates. (B) PBFI/AM-loaded J774A.1 macrophages were preincubated for 15 minutes in HBSS alone, or in HBSS buffer without glucose but containing 0.01% sodium azide and 10 mM 2DG. K^+^ efflux was monitored upon addition of 300 ng/ml of *Bp*-CyaA and the shown curves are representative of three independent experiments.

To address the relative contribution of cell surface retention of CyaA pores to the overall cytotoxic potency of low amounts of intact (AC^+^) CyaA, the natively fatty-acylated *Bp*-CyaA purified from *B. pertussis* 18323/pHSP9 was used [28]. *Bp*-CyaA exhibits about fourfold higher specific pore-forming (hemolytic) activity than the recombinant *rEc*-CyaA produced in *E. coli* ([11] and Figure S4). Therefore the use of *Bp*-CyaA allowed maximizing the observable amplitude of changes in cell permeabilizing and cytolytic capacity that would result from alterations of the rate of toxin pore removal from cell membrane. As indeed shown in Figure 12, as little as 100 ng/ml of *Bp*-CyaA promoted a detectable LDH release form J774A.1 cells already in 2 hours of incubation. Inhibition of endocytic uptake of *Bp*-CyaA with 2DG and sodium azide treatment then increased the cytolytic potency of *Bp*-CyaA by a factor of 2 to 3 (Figure 12A) and strongly increased the capacity of *Bp*-CyaA to permeabilize cells for K^+^ efflux, which become well observable already at 300 ng/ml of the toxin (Figure 12B).

## Discussion

**Figure 13:**
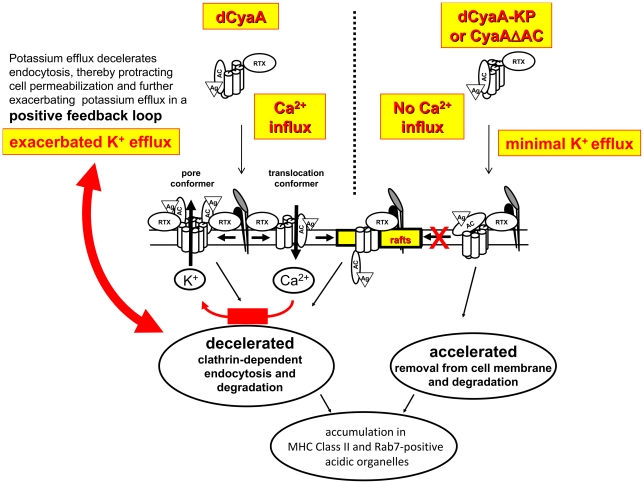
Model of the calcium influx-triggered positive feedback mechanism of self-exacerbating potassium efflux due to protracted cell permeabilization by toxin pores. Upon binding to the α_M_β_2_ integrin (CD11b/CD18), the pore precursor conformers of dCyaA oligomerize within the bulk phase of target cell membrane and permeabilize cells for efflux of cytosolic potassium ions. In parallel, toxin translocation precursors insert into cellular membrane and conduct extracellular Ca^2+^ into cell cytosol. Elevation of [Ca^2+^]*_i_* in the submembrane compartment results in activation of talin cleavage by calpain and liberates the receptor with bound toxin translocation precursor within target membrane for lateral relocation into lipid rafts, where the translocation of the AC domain across plasma membrane is completed [15]. The entry of Ca^2+^ provokes deceleration of clathrin-dependent endocytic uptake of CyaA, thereby delaying removal of toxin pores from the cytoplasmic membrane. Protracted persistence of CyaA pores within cellular membrane then triggers a self-amplifying (positive) feedback loop mechanism that exacerbates cell permeabilization. The more potassium leaks out form cells, the more the clathrin-dependent endocytic removal of toxin pores from cellular membrane is decelerated and the more is the cell permeabilization and potassium leakage exacerbated. In contrast, the mutated dCyaA-KP and the CyaAΔAC hemolysin variants of CyaA are unable to conduct calcium ions into cells and are thus rapidly removed from the cytosolic membrane of cells by a membrane macropinocytosis mechanism that directs the mutant toxoids for rapid proteolytic degradation.

We show here that by conducting Ca^2+^ ions across target cell membrane, CyaA decelerates its endocytic uptake and escapes from rapid macropinocytic removal from cell membrane and destruction in endosomes. By redirecting the toxin into a decelerated clathrin-dependent uptake pathway, the calcium-conducting activity of toxin translocation intermediates protracts toxin pore persistence within cytoplasmic membrane, thus extending phagocyte permeabilization and maximizing cytotoxic action of CyaA, as summarized in the model proposed in Figure 13.

This mechanism could be directly demonstrated here for the CyaA-AC^−^ toxoid and CyaA-ΔAC hemolysin variants that lack the AC enzyme activity and could be used at sufficiently high concentrations for live cell imaging. Imposing on CyaA-ΔAC a conformation that enabled it to conduct Ca^2+^ into cells, indeed, rescued the hemolysin from the macropinocytic pathway, and by decelerating its removal form cell membrane, it particularly enhanced the otherwise very low cell-permeabilizing and cytolytic capacity of CyaA-ΔAC.

Using intact toxin (AC^+^) purified form *B. pertussis* cells at close to physiologically relevant concentrations (200 ng/ml), we found that inhibition of endocytic uptake of *Bp*-CyaA enhanced its capacity to lyze cells. It was, however, not possible to design an experiment that would directly prove the role of calcium-induced deceleration of CyaA uptake in its cytotoxic action. Such demonstration would only be possible if inhibition of Ca^2+^ influx would leave the other cytotoxic activities of CyaA unaffected. This would then allow to test whether cytolytic activity of CyaA is reduced by acceleration of the endocytic uptake of the intact toxin. However, Ca^2+^ influx into cells is a prerequisite for CyaA relocation into lipid rafts and subsequent AC domain translocation into cell cytosol [15]. Therefore, blocking of Ca^2+^ influx by whatever means inevitably ablates also the major component of the cytotoxic activity of CyaA, namely the capacity of its AC enzyme to reach cell cytosol and catalyze unregulated dissipation of cytosolic ATP into cAMP to impair cellular signaling.

We have previously shown that upon initial binding of the CD11b/CD18 integrin, the CyaA toxin inserts into the membrane lipid bilayer as a translocation precursor, in which the segments of the AC domain cooperate with segments of the pore-forming domain in forming a novel calcium-conducting path across the phagocyte membrane that mediates influx of extracellular Ca^2+^ ions into cells [14], [15]. Activation of calpain by incoming Ca^2+^ then yields cleavage of the talin tether of CD11b/CD18 and liberates the toxin-receptor complex for recruitment into membrane lipid rafts [15]. This process appears to be entirely independent of the cAMP-generating capacity of CyaA in CD11b^+^ J774A.1 macrophage cells, as a quite comparable amplitude and even faster initial kinetics of calcium mobilization into cells is observed with the enzymatically inactive dCyaA variant (CyaA-AC^−^) than by intact CyaA [14], [15]. The results presented in this study then show that it is rather the permeabilization of cells for influx of Ca^2+^, than the toxin relocation into lipid rafts itself, which allows the toxoid to escape the rapid macropinocytic removal from cell surface with the cytoplasmic membrane. Moreover, lipid rafts are tiny microdomains in the membrane that would accommodate only limited amounts of the hemolysin, while a quantitative escape from rapid macropinocytosis of dCyaA-KP toxoid and its redirection for decelerated endocytosis was observed upon co-incubation with dCyaA (Figure 6A). This indicates that by permeabilizing cells for calcium influx, the AC-translocating CyaA conformers provoke also deceleration of endocytosis of the bystander CyaA pores that can form outside the rafts, in the bulk phase of cell membrane.

Such conclusion would also be in line with the observed opposing phenotypes of the CyaA-E509K+516K (CyaA-KK) and CyaA-E570Q+K860R (CyaA-QR) variants of CyaA. While the CyaA-KK exhibits a strongly enhanced specific pore-forming activity, its capacity to promote Ca^2+^ influx into cells is very low [14], [29]. On the opposite, the CyaA-QR toxin exhibits a very low capacity to form pores, to permeabilize cells and elicit K^+^ efflux, while it mediates normal levels of calcium influx into cells [6], [15]. This suggests that K^+^ efflux and Ca^2+^ influx are two parallel and independent processes that are associated with distinct conformational and/or oligomeric states of the two co-existing conformers of membrane-inserted CyaA.

In this respect, it is puzzling that binding of the 3D1 antibody to CyaA-ΔAC exacerbated at the same time its capacity to conduct calcium ions into cells, as well as its cell permeabilizing capacity. This would, perhaps, be best explained by the bystander effect mentioned above. Due to association-dissociation equilibrium, the antibody would lock only a fraction of membrane-inserted CyaA-ΔAC molecules into a calcium-conducting conformation. The other fraction of hemolysin molecules, not bound with 3D1, would hence be free to form the cell-permeabilizing oligomeric pores promoting K^+^ efflux, benefiting from the calcium-induced deceleration of the endocytic uptake of the hemolysin pores. It remains, nevertheless, to be conclusively shown that bridging of CyaA-ΔAC dimers by the bivalent 3D1 antibody does not also contribute to the enhanced cell permeabilization capacity and K^+^ efflux mediated by the CyaA-ΔAC hemolysin complexes with the antibody. 3D1 might, indeed, potentially stabilize the formed oligomeric pores against dissociation within the membrane. At present it can neither be formally excluded that the complex of membrane-inserted CyaA-ΔAC with 3D1 might be conducting at the same time the Ca^2+^ ions into cells and the K^+^ ions out of the cell. For intact CyaA the accumulated evidence strongly argues against such possibility (see above). Due to absence of the translocated AC domain, however, the 3D1-locked conformers of CyaA-ΔAC may be capable of oligomerization into pores enabling K^+^ efflux, or the calcium-conducting path formed by these conformers may be accessible for efflux of cytosolic K^+^ ions in the absence of the AC domain.

A particularly intriguing observation is that the Ca^2+^ influx and K^+^ efflux-promoting activities of the toxin synergize in manipulating the pathway and kinetics of CyaA endocytosis. Indeed, the decrease of intracellular potassium level was repeatedly shown to cause inhibition of clathrin-mediated endocytosis [30], [31]. Hence, the more the cell becomes permeabilized for efflux of K^+^, upon inhibition of macropinocytic uptake of toxin pores by incoming Ca^2+^, the more the potassium leakage from cells through toxin pores further decelerates the clathrin-dependent removal of CyaA pores from cell membrane. Such cooperation of Ca^2+^ influx and K^+^ efflux-mediating activities of CyaA would thus generate a positive feedback loop, exacerbating potassium depletion due to steadily increasing extent of cell membrane permeabilization by persisting and/or newly forming CyaA pores.

The above outlined positive feedback loop of potassium efflux was apparently operating under the used experimental conditions, since vesicles containing dCyaA were observed to accumulate as tightly attached to, or located just beneath the cell membrane, for over 20 minutes from toxoid addition (Figure 1 and Video S1). This shows that excision of clathrin-coated vesicles from cell membrane was inhibited and protraction of cell membrane permeabilization by the persisting pores then fed back into deceleration of endocytic uptake of dCyaA.

The enzymatically active CyaA could not be used in this study for analysis of endocytic trafficking of CyaA by live cell microscopy imaging, as at the high concentrations of labeled proteins (>1 µg/ml), required for this type of analyzes, the enzymatically active CyaA uncontrollably impairs intracellular trafficking by rapid depletion of ATP and induction of cell death [8], [9]. Indeed, the cytotoxic action of enzymatically active CyaA on CD11b^+^ phagocytes was documented repeatedly at doses lower than 10 ng/ml, where less than 1 ng/ml of active CyaA toxin quantitatively inhibits oxidative burst in neutrophils [32]. As low CyaA doses as 5 to 10 ng/ml elicit monocyte ruffling and a near-instant inhibition of CR3-mediated opsonophagocytosis or arrest of the fluid-phase uptake, respectively. This appears to be due to cAMP signaling-mediated inhibition of the small GTPase RhoA and possibly of the PI3 kinase [21]. The results obtained here with the toxoids appear, nevertheless, to be relevant also to the understanding of endocytosis and of cytotoxic action of the enzymatically active CyaA. By using a newly developed FACS assay for cell surface accessibility of bound CyaA, we could approach here the kinetics of endocytic removal of intact CyaA from cell surface at close to physiologically low toxin concentrations (200 ng/ml). Under such conditions, the receptor-bound CyaA was found to be progressively removed from cell surface over 15 minutes of incubation with about one quarter of toxin molecules escaping the uptake into endosomes and remaining exposed on the surface of cell membrane. This endocytic uptake of intact CyaA from cell membrane was noticeably slowed down in the presence of a five-fold excess of enzymatically inactive CyaA-AC^−^ molecules that promoted Ca^2+^ influx into cells and K^+^ efflux *in trans*. Moreover, inhibition of the endocytic uptake through inhibition of ATP re-synthesis strongly enhanced the capacity of native *Bp*-CyaA toxin to permeabilize and lyze cells already at as low toxin concentrations as 200 ng/ml. This points towards a more important contribution of the pore-forming activity to the overall cytotoxic action of CyaA than previously recognized [8], [9].

Enzymatically inactive but pore-forming CyaA-AC^−^ toxoids have been extensively used over the past 18 years for delivery of AC-inserted CD8^+^ and CD4^+^ T cell epitopes from viruses, mycobacteria or tumors into the MHC class I and II-restricted antigen presentation pathways of CD11b-expressing dendritic cells [33]–[36]. As dCyaA-based vaccines for cancer immunotherapy are currently in phase I of clinical studies (www.genticel.com), the here reported insight into dCyaA endocytosis and trafficking paves the way towards deciphering of the efficacy of T cell vaccine delivery by CyaA toxoids. Moreover, an endotoxin-free CyaA-AC^−^ (dCyaA) toxoid was recently observed to alter the expression levels of a dozen of genes involved in innate immune response signaling in bone marrow-derived macrophages [37]. This is likely due to the capacity of the toxoid to promote Ca^2+^ influx into cells and permeabilize cells for K^+^ efflux. A long-sought evidence for a role of the pore-forming capacity of CyaA in *Bordetella pertussis* infection has, indeed, been recently reported by Dunne and coworkers [29]. This study showed that by eliciting K^+^ efflux from dendritic cells, and perhaps some other CD11b-expressing phagocytes, the pore-forming activity of CyaA contributes to activation of the NALP3 inflammasome and thereby to induction of innate IL-1β response, which supports the clearance of *Bordetella* bacteria at later stages of infection. The results reported herein show that the capacity of CyaA to permeabilize cells for K^+^ efflux depends on the capacity of the toxin to promote Ca^2+^ influx into cells and escape the rapid macropinocytic removal from target cell membrane. This reveals a further layer of sophistication of CyaA action on host cells, thus underpinning the key role of this toxin with multiple ‘talents’ in the virulence of *Bordetellae* in mammals.

## Materials and Methods

### Antibodies and reagents

Mouse monoclonal antibody (MAb) anti-CD71 (clone MEM-189) was from Exbio (Vestec, Czech Republic), anti-NTAL MAb (clone NAP-08) was a generous gift from Pavla Angelisova (Institute of Molecular Genetics, Prague, Czech Republic) and anti-CyaA MAbs clone 9D4 and 3D1 were kindly provided by Erik L. Hewlett (University of Virginia School of Medicine, Charlottesville, USA). Fura-2/AM, PBFI/AM, Alexa Fluor 488, LysoTracker-red DND-99, DAPI (4′-6-diamidino-2-phenylindole) and Mowiol were purchased from Molecular Probes (Eugene, OR). Transferrin coupled to Dyomics 547, BSA-Dyomics 547 and mouse monoclonal antibody against α-tubulin (TU-01, IgG1 isotype control) were from Exbio (Vestec, Czech Republic). Ionomycin, valinomycin, nigericin, wortmannin, chlorpromazine, dynasore, 2-deoxy-D-glucose, sodium azide, chloroquine and Pluronic F-127 were purchased from Sigma (St. Louis, MO). Complete Mini protease inhibitors cocktail was purchased from Roche (Basel, Switzerland). NHS-Sulfo-LC-Biotin was purchased from Pierce (Rockford, IL, USA). The SIINFEKL peptide corresponding to the CD8^+^ T-cell epitope encompassing the chicken ovalbumin (OVA) residues 257–264 and to the CD4^+^ T-cell epitope NGKLIAYPIAVEALS peptide corresponding to the *Escherichia coli* MalE protein (residues 100–114 [18]), respectively, were purchased from Neosystem (Strasbourg, France).

### Construction, production and purification of CyaA-derived proteins

Construction of dCyaA was described earlier [18]. dCyaA-OVA_258–276_, harboring the VQLTGLEQLESIINFEKLTEWTSS NVMEERKIKVYLPRIVH peptide from hen egg ovalbumin protein (OVA, residues 249–284) and carrying the MHC Class II sequence IINFEKLTEWTSSNVMEER (OVA_258–276_) was constructed according to the standard protocols by insertion of a corresponding double-stranded synthetic oligonucleotide between codons 107 and 108 of the *cyaA* gene (Holubova *et al.*, Infect. Immun. doi:10.1128/IAI.05711-11, published online ahead of print on 3 January 2012). The corresponding CyaA protein variants, harboring the E570K and E581P substitutions, dCyaA-KP and dCyaA-OVA_258–276_-KP were constructed by recombination with previously described plasmid constructs [18], [38]. Toxoid constructs were genetically detoxified by insertion of the dipeptide sequence GlySer between residues 188 and 189, thus disrupting the ATP binding site of the enzyme [39]. CyaA derivatives were produced using *E. coli* strain XL1-Blue (Stratagene, La Jolla, CA) in the presence of CyaC acyltransferase produced from the same plasmid and were purified by chromatography on DEAE-Sepharose and Phenyl-Sepharose as described earlier [40]. Enzymatically active CyaA (r*Ec*-CyaA or CyaA) produced in *E. coli* XL1-Blue and *Bp*-CyaA produced in *B. pertussis* strain 18323/ pHSP9 [28] were purified from urea extracts by combination of chromatography on DEAE-Sepharose and calmodulin agarose. All experiments were repeated with proteins from at least two independent preparations.

### Macrophage binding, cell lytic capacities and determination of intracellular cAMP

J774A.1 macrophages (3×10^5^) were incubated in D-MEM with 200 ng/ml of CyaA-biotin for 5 min at 37°C, prior to removal of unbound toxin by three washes in cold D-MEM medium. Cells were lyzed with 0.1% Triton X-100 for determination of cell-bound AC activity. Toxin-induced lysis of J774A.1 cells was determined using the CytoTox 96 kit assay (Promega, Madison, USA) as the amount of lactate dehydrogenase released from 2×10^5^ cells in 2 hours of incubation with CyaA at 37°C in D-MEM. 10^5^ J774A.1 cells were incubated with different concentrations of the CyaA derived constructs for 30 minutes in D-MEM medium without FCS (fetal calf serum, Life Technologies, Gaithersburg, USA). The reaction was stopped by addition of 0.2% Tween-20 in 50 mM HCl and samples were boiled for 15 min to denature cellular proteins. The samples were neutralized by addition of 150 mM unbuffered imidazol and concentration of cAMP was determined by a competition imunoassay performed as previously described [8].

### Protein labeling

Toxoids were labeled with the amine-reactive Alexa Fluor 488 or Dyomics 647 dyes upon binding to Phenyl-Sepharose during the last purification step in 0.1 M NaHCO_3_ pH 8.3 at room temperature for 1 hour. Unreacted dye was washed-out from the resin with 50 mM Tris-HCl buffer (pH 8.0) and labeled toxoids were eluted from Phenyl-Sepharose in TUE buffer (Tris 50 mM, 8M Urea, 2 mM EDTA, pH 8.0). The molar ratio of protein∶dye was approximately 1∶4 for all toxoid preparations. On-column biotinylation of r*Ec*-CyaA toxin was performed after the DEAE-Sepharose purification step using NHS-Sulfo-LC-Biotin (Pierce, Rockford, IL, USA) to reach a biotin∶toxin molar ratio of 20∶1. Biotin coupling was at room temperature and was stopped after 40 min by washing of the resin with 15 ml of 50 mM Tris-HCl, pH 8.0, and then extensively with PBS (phosphate-buffered saline). Purified biotinylated toxin was then eluted with 50 mM HEPES, 8 M urea, and 2 mM EDTA.

### Cell cultures, growth conditions and handling of cells

J774A.1 cells (ATCC TIB 67) were maintained in RPMI 1640 medium containing 10% FCS and antibiotic/antimycotic solution (0.1 mg/ml streptomycin, 1000 U/ml penicillin and 0.25 µg/ml amphotericin (Sigma, St. Louis, MO)). For all experiments the RPMI 1640 medium used for cell cultivation was replaced by Dulbecco's modified Eagle's medium (D-MEM) containing 1.9 mM Ca^2+^ without FCS, to avoid uncontrollable chelation of calcium ions by the phosphate ions contained in RPMI 1640 medium, as calcium is required for CyaA activity. Bone marrow derived dendritic cells (BMDCs) from MHC Class II/EGFP knock-in mouse [41] were flushed from the femur bone marrow cavity with PBS/2.5% FCS, plated at approx. 10^6^ cells/well in 2 ml of DMEM/25 mM HEPES/10% FCS without phenol red but containing 5 ng/ml GM-CSF (Sigma, St. Louis, MO). Cells were cultured on 25-mm circular cover slips, with media replacement every second day. Non-adherent cells were removed by gentle washing on days 2 and 4. BMDCs from conventional 6- to 8-week-old female C57BL/6 mice were obtained from femoral and tibial bones and cells were cultured in RPMI 1640 medium supplemented with 10% FCS, 20 ng/ml GM-CSF and antibiotic/antimycotic solution for 7 days, as previously described by [42].

### Transfection of RAW 264.7 macrophages

RAW 264.7 (ATCC TIB 71) cells (5×10^4^) grown on coverslips were maintained in RPMI medium supplemented with 10% FCS and transfected with pEGFP constructs bearing cDNA encoding Eps-15 (DIII) or Rab-7 (kind gift of J. Forstova, Charles University, Prague) using a FuGENE-6 transfection reagent (Roche).

### Isolation of detergent resistant membrane and western blotting

Detergent resistant membranes (coalesced lipid rafts) were separated by flotation in discontinuous sucrose density gradients. J774A.1 cells (2×10^7^) were washed with prewarmed DMEM and incubated with 500 ng of CyaA-derived proteins at 37°C for 10 min. Cells were washed with ice-cold PBS, scraped from the Petri dish and extracted at 4°C in 200 µl of TBS buffer (20 mM Tris-HCl, pH 7.5, 150 mM NaCl) containing 1% Triton X-100, 1 mM EDTA, 10 mM NaF and a Complete Mini protease inhibitor cocktail (Roche) for 60 min. The lyzate was clarified by centrifugation at 250× *g* for 5 min and the post-nuclear supernatant was mixed with equal volume of 90% sucrose in TBS. The suspension was placed at the bottom of a centrifuge tube and overlaid with 2.5 ml of 30% sucrose and 1.5 ml of 5% sucrose in TBS. Buoyant density centrifugation was performed at 150,000× *g* in a Beckman SW60Ti rotor for 16 h at 4°C. Fractions of 0.5 ml were removed from the top of the gradient. For immunodetection, the separated proteins were transferred onto Immobilon-P membrane, blocked with 5% non-fat milk powder in TBST buffer (20 mM Tris-HCl, pH 7.5, 150 mM NaCl, 0.05% Tween-20) and probed with the indicated mouse monoclonal antibody. CyaA toxoids were recognized in Western blots by the 9D4 antibody binding to the C-terminal RTX repeats of CyaA. The signal was developed using a secondary peroxidase-conjugated sheep anti-mouse IgG and chemiluminescent detection system (SuperSignal West Femto Maximum Sensitivity Substrate chemiluminescence reagent kit, Pierce, Rockford, IL).

### Cell treatment and imaging

J774A.1 mouse monocytes were grown on glass coverslips to subconfluence in the absence of the pH indicator (to avoid cellular autofluorescence). Cells were incubated with Alexa Fluor 488-labeled toxoid variants in serum free D-MEM medium at 37°C in the presence of 10 µg/ml transferrin-Dyomics 547 or in the presence of 50 µg/ml BSA-Dyomics 547 (Exbio, Czech Republic). For *in vivo* imaging, J774A.1 cells grown on glass bottom microwell dishes (MatTek, USA) were incubated in the presence of labeled toxoids at 37°C in HBSS medium alone, or in the presence of inhibitors. BMDCs from day 5 were incubated for 2 hours with 1 µg/ml of Dyomics-647-labeled toxoid variants at 37°C in DMEM without serum. For simultaneous visualization of early and recycling endocytic compartments, DCs were co-incubated with 25 µg/ml transferrin-Dyomics-547 for the last 30 minutes. Cells were fixed (4% PFA in PBS) and mounted in Mowiol. Images were captured using a Leica confocal microscope TCS SP2 (Wetzlar, Germany) or a Cell^R^ Imaging Station (Olympus, Hamburg, Germany) based on Olympus IX 81 fluorescence microscope.

### Statistical analysis of colocalization

For colocalization analysis the 3D stack of desired colour channels was acquired comprising most of the cell volume (usually 10–15 planes in the z-axis). Analysis was performed using a macro in WCIF ImageJ software. Special care was taken of the pixel shift between individual colour channels (calibration with fluorescent beads). The threshold levels for each image and channel was found using “Huang dark” method. Individual cells were selected (as ROI) and colocalization analysis was performed using an ImageJ plug-in (http://www.uhnresearch.ca /facilities/wcif/imagej/colour_analysis.htm). Pearson's correlation coefficients for each image (individual cell recorded in all z-axis planes) were calculated for the given channels. Usually about 60 cells were analyzed and average Pearson's correlation coefficient (P.c.c.) of all z-axis planes ± standard deviation was calculated. A value of 1 represents perfect correlation, zero represents random localization.

### Quantification of endosomes

For this purpose a script based on WCIF ImageJ software was used (see Figure S2 for detailed description).

### Measuring of intracellular concentration of ions

Calcium influx into cells was measured as previously described [14]. Briefly, the J774A.1 cells were loaded with 3 µM Fura-2/AM (Molecular Probes) at 25°C for 30 min and time course of calcium entry into cells after addition of CyaA-derived proteins was determined as ratio of fluorescence excited at 340/380 nm. Fluorescence measurement of cytosolic K^+^ was performed as described before [6]. Briefly, J774A.1 cells were loaded with 9.5 µM PBFI/AM (Molecular Probes) for 30 min at 25°C in the presence of 0,05% (w/w) Pluronic F-127 (Sigma-Aldrich) in the dark. Fluorescence intensity of PBFI (excitation wavelength 340, emission wavelengths 450 and 510 nm) was recorded, ratio of these intensities are shown in the graphs. Calibration experiments were performed in solutions containing 50 mM HEPES (pH 7.2), with varying concentrations of potassium acetate (10, 30, 60, or 140 mM) and sodium acetate (5, 85, 115, or 135 mM). Cellular plasma membrane was permeabilized for potassium ions and protons by valinomycin and nigericin (3 µM; Sigma-Aldrich) for 30 min.

### Antigen presentation assays

The H-2^b^-restricted T cell hybridoma CRMC3, recognizing the NGKLIAYPIAVEALS epitope of the MalE protein from *E. coli* (MalE_100–114_, abbreviated MalE), and the I-A^b^-restricted T cell hybridoma MF2.2D9, recognizing the IINFEKLTEWTSSNVMEER epitope of ovalbumin (OVA_258–276_), respectively, were used. BMDC (10^5^ cells/well) were pulsed with proteins or peptides for 4–5 hours, followed by medium disposal and cultivation with CRMC3 hybridoma (10^5^ cells/well) for 18 hours or the MF2.2D9 hybridoma (5×10^4^) for 14 hours, respectively. Prior to CRMC3 hybridoma addition the BMDCs were washed with PBS. After cell co-incubation, cultures were frozen for at least 2 hours at −80°C. T cell stimulation was monitored by determination of IL-2 amounts released into culture supernatants using two alternative methods. CRMC3 culture supernatants (100 µl) were added to cultures of the IL-2-dependent CTL-L cell line (10^4^ cells/well, final volume 200 µl) for 48 hours, followed by addition of [^3^H]thymidine (50 µCi/ml; Perkin Elmer, Courtabeuf, France). Cells were harvested 6 hours later using an automated cell harvester (Molecular Devices, Lier, Norway) and the incorporated thymidine was determined by scintillation counting. The concentration of IL-2 released into MF2.2D9 cell culture supernatants was determined using a sandwich ELISA with noncompeting pairs of anti–IL-2 mAbs (JES6-1A12 and biotinylated JES6-5H4, both from BD Pharmingen, San Diego, CA, USA.

## Supporting Information

Figure S1 **CyaA constructs with the deletions in the hydrophobic domain failed to deliver their MalE protein for MHC Class II-restricted presentation by dendritic cells, despite a preserved capacity to bind the CD11b/CD18 receptor.** (A) Schematic depiction of the various CyaA variants carrying MalE protein in place of the AC domain. The sequence encoding maltose binding protein (MalE) was amplified from a genomic DNA of *E. coli* K-12 by PCR and cloned into the pET28b vector (Novagen) for the expression of MalE-C-His, or into the pT7CT7ACT1 plasmid (J. Holubova-Hejnova, unpublished) for the expression of MalE-RTX. Plasmids for the production of deletion variants of MalE-RTX (MalE-RTX-Δ374–647, MalE-RTX-Δ647–855 and MalE-RTX-Δ374–855) were constructed using restriction endonucleases and standard cloning techniques. In the names of the CyaA variants the symbol Δ is followed by the numbers of the first and last amino acid residues of the deleted parts of CyaA. Deleted portions are indicated by the lines. MalE protein is represented as a grey bar. All constructs were expressed in *E. coli* BL-21 and purified by chromatography on DEAE Sepharose. MalE-C-His was purified by combination of chromatography on Amylose, Ni-NTA-Agarose and Phenyl Sepharose. (B) Deletions in the hydrophobic domain of CyaA partially impair CD11b/CD18 binding. CHO-CD11b/CD18 cells were preincubated with different concentrations of deletion variants of CyaA carrying MalE protein in place of the AC domain (1–373) for 30 minutes on ice. Then, CyaA-biotin (30 nM) was added and the amount of surface-bound CyaA-biotin was determined by streptavidine-PE and flow cytometry. As a negative control, the entire MalE-C-His protein unfused to CyaA was used. Results are expressed as % of CyaA-biotin in the presence of the various CyaA competitors determined as (bound CyaA-biotin in the sample)/(maximal CyaA-biotin binding in the absence of competitor CyaA)×100%. The results shown are representative of two independent experiments performed in duplicates. (C) Deletions in the hydrophobic domain of CyaA completely abolish delivery of the MalE protein into the MHC Class II antigen presentation pathway. BMDCs from C57BL/6 mice were incubated for 5 hours with various concentrations of dCyaA, MalE-RTX or deletion toxoid variants. After incubation, BMDCs were washed and incubated with anti-MalE class II restricted specific hybridoma CRMC3 for 18 hours. The amounts of IL-2 secreted by cell hybridoma during the 18 hours culture were monitored using the IL-2-dependent CTL-L cell line. The results are expressed in cpm.
(TIF)

Figure S2 **Quantification of internalized endosomes. For this purpose a script based on WCIF ImageJ (v. 143 g) software was used (** http://rsb.info.nih.gov/ij **,** http://www.uhnresearch.ca/facilities/wcif/imagej **).** All images were converted to monochromatic 8-bit scale and processed in format 672×512 pixels. A copy of original image was smoothed by Gaussian blur (radius 2.5 pixels). This blurred image was subtracted from the original image and the result served as template for subsequent particle (endosomal) recognition. Threshold intensity was found by “MaxEntropy dark” automatic algorithm and all particles brighter than the threshold and larger than 3 pixels were recognized and recorded. For visual inspection, original image was shown in green and recognized endosomes in red (see picture). Due to colocalization of recognized endosomes with the higher intensity in the original image the endosomes are yellow. Endosomes within cell were recognized by this approach (left panel) whereas most of the endosomes remaining attached to the bright membrane cell membrane, or localized in its close proximity, remained unrecognized and were not counted (right panel). This algorithm was used repeatedly for all images of the time series. Subsequently, individual cells in the images were analysed semi-manually, in order to obtain average numbers of recognized endosomes per cell. These values are plotted in the main text Figures. Each plot shows one representative experiment (n = 3) and error bars correspond to standard deviations of endosomal numbers for this experiment, including values form 20–40 cells.
(TIF)

Figure S3 **Neither dCyaA nor dCyaA-KP colocalize with caveolin-1.** J774A.1 cells were incubated with 5 µg/ml of Alexa Fluor 488-labeled dCyaA or dCyaA-KP at 37°C. After 5 minutes, cells were washed in cold PBS and fixed by 4% PFA. Caveolin was labeled with anti-Caveolin-1 antibody (N-20, rabbit polyclonal, Santa Cruz) and anti-rabbit IgG-Alexa 594 (Molecular Probes). Nuclei were stained with DAPI (2 µg/ml, Molecular Probes). Samples were observed using an Olympus Cell^R^ IX 81 microscope with a 100× oil immersion objective (N.A. 1.3). Values of the Pearson's correlation coefficients for compared channels were 0.095 and 0.060 for dCyaA and dCyaA-KP, respectively.
(TIF)

Figure S4 **Comparison of specific hemolytic activities of native and recombinant CyaA.** Sheep erythrocytes (5×10^8^/ml) in Tris 50 mM, NaCl 150 mM, CaCl_2_ 2 mM, pH 7.4 were incubated with 2 µg/ml of recombinant (r*Ec-*CyaA) CyaA from *E. coli* or with CyaA purified from and overproducing *B. pertussis* 18323/pHSP9 strain (*Bp-*CyaA) at 37°C. Hemolytic activity was measured as the amount of released hemoglobin by photometric determination (A_541_). The results represent the average of values obtained in two independent experiments performed in duplicates.
(TIF)

Video S1 **Endocytosis of Dyomics 647-labeled dCyaA.** J774A.1 cells were grown on glass bottom microwell dishes and incubated for 20 minutes at 37°C with 5 µg/ml of Dyomics 647-labeled dCyaA. Toxoid endocytosis was followed for 3 to 20 minutes of incubation by *in vivo* cell imaging using an Olympus Cell^R^ IX 81 microscope equipped with a 60×/1.35 oil objective (UPlanSApo). Video sequences were processed using ImageJ software, RGB 512×512 pixels, MPEG-4 video, Xvid compression, 2 frames per second.
(AVI)

Video S2 **Endocytosis of Alexa Fluor 488-labeled dCyaA-KP.** J774A.1 cells grown on glass bottom microwell dishes were incubated for 20 minutes at 37°C with 5 µg/ml of Alexa Fluor 488-labeled dCyaA-KP. Toxoid endocytosis was followed for 3 to 20 minutes of incubation by *in vivo* cell imaging using an Olympus Cell^R^ IX 81 microscope equipped with a 60×/1.35 oil objective (UPlanSApo). Video sequences were processed using ImageJ software, RGB 512×512 pixels, MPEG-4 video, Xvid compression, 2 frames per second.
(AVI)

Video S3 **Endocytosis of Dyomics 647-labeled dCyaA-KP in the presence of ionomycin.** J774A.1 cells grown on glass bottom microwell dishes were incubated for 20 minutes at 37°C with 5 µg/ml of Dyomics 647-labeled dCyaA-KP in the presence of 10 µM ionomycin. Toxoid endocytosis was followed for 3 to 20 minutes of incubation by *in vivo* cell imaging using an Olympus Cell^R^ IX 81 microscope equipped with a 60×/1.35 oil objective (UPlanSApo). Video sequences were processed using ImageJ software, RGB 512×512 pixels, MPEG-4 video, Xvid compression, 2 frames per second.
(AVI)

Video S4 **Endocytosis of Dyomics 647-labeled dCyaA-KP in the presence of Alexa Fluor 488-labeled dCyaA with J774A.1 cells grown on glass bottom microwell dishes were incubated for 20 minutes at 37°C with 5 µg/ml of 1∶1 mix of Alexa Fluor 488-labeled dCyaA with Dyomics 647-labeled dCyaA-KP.** Toxoid endocytosis was followed for 3 to 20 minutes of incubation by *in vivo* cell imaging using an Olympus Cell^R^ IX 81 microscope equipped with a 60×/1.35 oil objective (UPlanSApo). Video sequences were processed using ImageJ software, RGB 512×512 pixels, MPEG-4 video, Xvid compression, 2 frames per second.
(AVI)

Video S5 **Endocytosis of Alexa Fluor 488-labeled CyaA-ΔAC.** J774A.1 cells grown on glass bottom microwell dishes were incubated for 20 minutes at 37°C with 5 µg/ml of Alexa Fluor 488-labeled CyaA-ΔAC. Toxoid endocytosis was followed for 3 to 20 minutes of incubation by *in vivo* cell imaging using an Olympus Cell^R^ IX 81 microscope equipped with a 60×/1.35 oil objective (UPlanSApo). Video sequences were processed using ImageJ software, RGB 512×512 pixels, MPEG-4 video, Xvid compression, 2 frames per second.
(AVI)

Video S6 **Endocytosis of Alexa Fluor 488-labeld CyaA-ΔAC in the presence of 2DG and azide.** J774A.1 cells grown on glass bottom microwell dishes were incubated for 20 minutes at 37°C with 5 µg/ml of Alexa Fluor 488-labeled CyaA-ΔAC in the presence of 2DG (10 mM) and sodium azide (0.01%). Toxoid endocytosis was followed for 3 to 20 minutes of incubation by *in vivo* cell imaging using an Olympus Cell^R^ IX 81 microscope equipped with a 60×/1.35 oil objective (UPlanSApo). Video sequences were processed using ImageJ software, RGB 512×512 pixels, MPEG-4 video, Xvid compression, 2 frames per second.
(AVI)
