# Supplementary figures and images for: Calcium Influx Rescues Adenylate Cyclase-Hemolysin from Rapid Cell Membrane Removal and Enables Phagocyte Permeabilization by Toxin Pores
Source: PLoS Pathog. 2012 Apr 5;8(4):e1002580. doi: 10.1371/journal.ppat.1002580 (PMC3320606; doi:10.1371/journal.ppat.1002580)

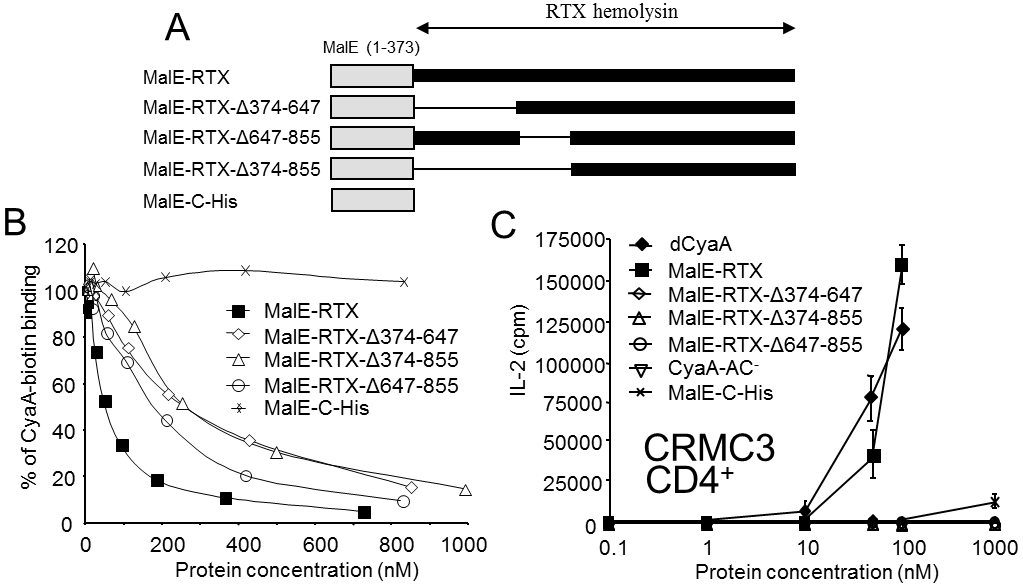

Supplement: Figure S1 — CyaA constructs with the deletions in the hydrophobic domain failed to deliver their MalE protein for MHC Class II-restricted presentation by dendritic cells, despite a preserved capacity to bind the CD11b/CD18 receptor. (A) Schematic depiction of the various CyaA variants carrying MalE protein in place of the AC domain. The sequence encoding maltose binding protein (MalE) was amplified from a genomic DNA of E. coli K-12 by PCR and cloned into the pET28b vector (Novagen) for the expression of MalE-C-His, or into the pT7CT7ACT1 plasmid (J. Holubova-Hejnova, unpublished) for the expression of MalE-RTX. Plasmids for the production of deletion variants of MalE-RTX (MalE-RTX-Δ374–647, MalE-RTX-Δ647–855 and MalE-RTX-Δ374–855) were constructed using restriction endonucleases and standard cloning techniques. In the names of the CyaA variants the symbol Δ is followed by the numbers of the first and last amino acid residues of the deleted parts of CyaA. Deleted portions are indicated by the lines. MalE protein is represented as a grey bar. All constructs were expressed in E. coli BL-21 and purified by chromatography on DEAE Sepharose. MalE-C-His was purified by combination of chromatography on Amylose, Ni-NTA-Agarose and Phenyl Sepharose. (B) Deletions in the hydrophobic domain of CyaA partially impair CD11b/CD18 binding. CHO-CD11b/CD18 cells were preincubated with different concentrations of deletion variants of CyaA carrying MalE protein in place of the AC domain (1–373) for 30 minutes on ice. Then, CyaA-biotin (30 nM) was added and the amount of surface-bound CyaA-biotin was determined by streptavidine-PE and flow cytometry. As a negative control, the entire MalE-C-His protein unfused to CyaA was used. Results are expressed as % of CyaA-biotin in the presence of the various CyaA competitors determined as (bound CyaA-biotin in the sample)/(maximal CyaA-biotin binding in the absence of competitor CyaA)×100%. The results shown are representative of two independent experiments [file ppat.1002580.s001.tif]

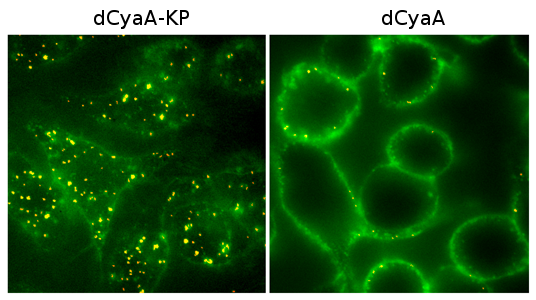

Supplement: Figure S2 — Quantification of internalized endosomes. For this purpose a script based on WCIF ImageJ (v. 143 g) software was used ( http://rsb.info.nih.gov/ij , http://www.uhnresearch.ca/facilities/wcif/imagej ). All images were converted to monochromatic 8-bit scale and processed in format 672×512 pixels. A copy of original image was smoothed by Gaussian blur (radius 2.5 pixels). This blurred image was subtracted from the original image and the result served as template for subsequent particle (endosomal) recognition. Threshold intensity was found by “MaxEntropy dark” automatic algorithm and all particles brighter than the threshold and larger than 3 pixels were recognized and recorded. For visual inspection, original image was shown in green and recognized endosomes in red (see picture). Due to colocalization of recognized endosomes with the higher intensity in the original image the endosomes are yellow. Endosomes within cell were recognized by this approach (left panel) whereas most of the endosomes remaining attached to the bright membrane cell membrane, or localized in its close proximity, remained unrecognized and were not counted (right panel). This algorithm was used repeatedly for all images of the time series. Subsequently, individual cells in the images were analysed semi-manually, in order to obtain average numbers of recognized endosomes per cell. These values are plotted in the main text Figures. Each plot shows one representative experiment (n = 3) and error bars correspond to standard deviations of endosomal numbers for this experiment, including values form 20–40 cells. (TIF) [file ppat.1002580.s002.tif]

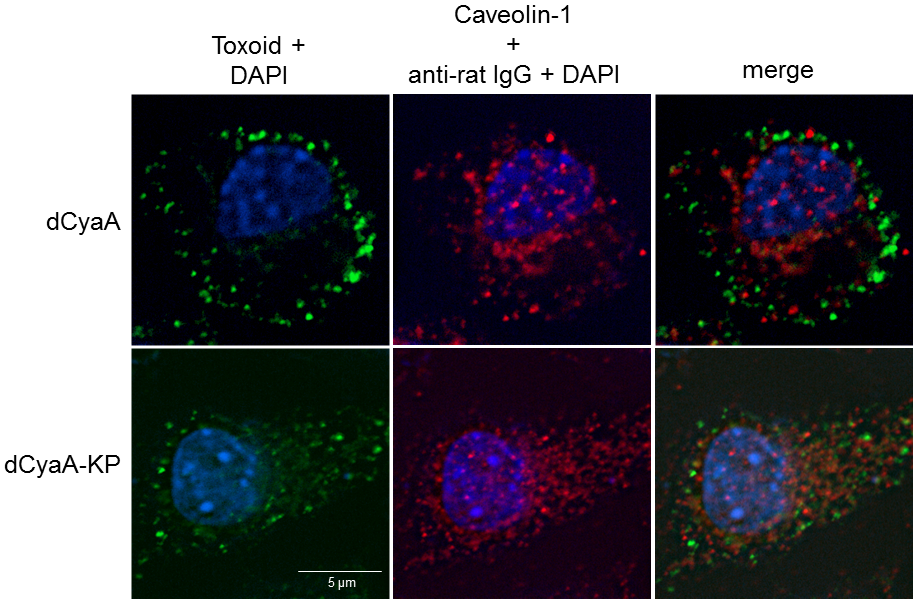

Supplement: Figure S3 — Neither dCyaA nor dCyaA-KP colocalize with caveolin-1. J774A.1 cells were incubated with 5 µg/ml of Alexa Fluor 488-labeled dCyaA or dCyaA-KP at 37°C. After 5 minutes, cells were washed in cold PBS and fixed by 4% PFA. Caveolin was labeled with anti-Caveolin-1 antibody (N-20, rabbit polyclonal, Santa Cruz) and anti-rabbit IgG-Alexa 594 (Molecular Probes). Nuclei were stained with DAPI (2 µg/ml, Molecular Probes). Samples were observed using an Olympus CellR IX 81 microscope with a 100× oil immersion objective (N.A. 1.3). Values of the Pearson's correlation coefficients for compared channels were 0.095 and 0.060 for dCyaA and dCyaA-KP, respectively. (TIF) [file ppat.1002580.s003.tif]

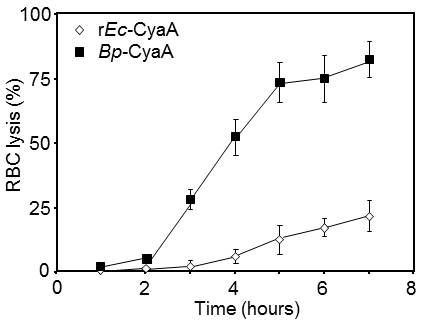

Supplement: Figure S4 — Comparison of specific hemolytic activities of native and recombinant CyaA. Sheep erythrocytes (5×10^8^/ml) in Tris 50 mM, NaCl 150 mM, CaCl2 2 mM, pH 7.4 were incubated with 2 µg/ml of recombinant (rEc-CyaA) CyaA from E. coli or with CyaA purified from and overproducing B. pertussis 18323/pHSP9 strain (Bp-CyaA) at 37°C. Hemolytic activity was measured as the amount of released hemoglobin by photometric determination (A541). The results represent the average of values obtained in two independent experiments performed in duplicates. (TIF) [file ppat.1002580.s004.tif]
